# Vulnerable newborn types: Analysis of population‐based registries for 165 million births in 23 countries, 2000–2021

**DOI:** 10.1111/1471-0528.17505

**Published:** 2023-05-08

**Authors:** Lorena Suárez‐Idueta, Judith Yargawa, Hannah Blencowe, Ellen Bradley, Yemisrach B. Okwaraji, Veronica Pingray, Luz Gibbons, Adrienne Gordon, Kara Warrilow, Enny S. Paixao, Ila Rocha Falcão, Sarka Lisonkova, Qi Wen, Francisco Mardones, Raúl Caulier‐Cisterna, Petr Velebil, Jitka Jírová, Erzsebet Horváth‐Puhó, Henrik Toft Sørensen, Luule Sakkeus, Lili Abuladze, Mika Gissler, Mohammad Heidarzadeh, Maziar Moradi‐Lakeh, Khalid A. Yunis, Ayah Al Bizri, Shamala D. Karalasingam, Ravichandran Jeganathan, Arturo Barranco, Lisa Broeders, Aimée E. van Dijk, Luis Huicho, Hugo Guillermo Quezada‐Pinedo, Kim Nail Cajachagua‐Torres, Fawziya Alyafei, Mai AlQubaisi, Geum Joon Cho, Ho Yeon Kim, Neda Razaz, Jonas Söderling, Lucy K. Smith, Jennifer Kurinczuk, Estelle Lowry, Neil Rowland, Rachael Wood, Kirsten Monteath, Isabel Pereyra, Gabriella Pravia, Eric O. Ohuma, Joy E. Lawn, the National Vulnerable Newborn Prevalence Collaborative Group and Vulnerable Newborn Measurement Core Group, Vicki Flenady, Harriet Lawford, Gabriela Cormick, Jose Belizan, Carlos Guevel, Mauricio Lima Barreto, José Acuña, Narjes Khalili, Pascale Nakad, Nurakman Binti Baharum, Jesus Felipe Gonzalez Roldan, Sonia Lopez Alvarez, Wilmer Cristobal Guzman‐Vilca, Carla Tarazona‐Meza, Rodrigo M. Carrillo‐Larco, Tawa O. Olukade, Hamdy A. Ali, Bradley N. Manktelow, Ruth J. Matthews, Elizabeth Draper, Alan Fenton, Celina Davis, Samantha Clarke, Robert E. Black, Joanne Katz, Daniel Erchick, Elizabeth Hazel, Mike Diaz, Anne C. C. Lee

**Affiliations:** ^1^ Mexican Society of Public Health Mexico City Mexico; ^2^ Maternal, Adolescent, Reproductive & Child Health (MARCH) Centre London School of Hygiene & Tropical Medicine London UK; ^3^ Department of Mother & Child Health Institute for Clinical Effectiveness and Health Policy Buenos Aires Argentina; ^4^ Faculty of Medicine and Health University of Sydney Sydney New South Wales Australia; ^5^ Centre for Research Excellence in Stillbirth, MRI‐UQ Mater Research Institute, The University of Queensland Brisbane Queensland Australia; ^6^ Centre of Data and Knowledge Integration for Health (CIDACS) Instituto Gonçalo Moniz, Fiocruz Bahia, Fundação Oswaldo Cruz Salvador Brazil; ^7^ Department of Obstetrics & Gynaecology University of British Columbia Vancouver British Columbia Canada; ^8^ Pontificia Universidad Católica de Chile Santiago Chile; ^9^ Department of Obstetrics and Gynaecology Institute for the Care of Mother and Child Prague Czech Republic; ^10^ Department of Data Analysis Institute of Health Information and Statistics of the Czech Republic Prague Czech Republic; ^11^ Department of Clinical Epidemiology Aarhus University Aarhus N Denmark; ^12^ School of Governance, Law and Society, Estonian Institute for Population Studies Tallinn University Tallinn Estonia; ^13^ Department of Knowledge Brokers THL Finnish Institute for Health and Welfare Helsinki Finland; ^14^ Department of Paediatrics Alzahra Hospital Tabriz Iran; ^15^ Department of Community Medicine, Preventive Medicine and Public Health Research Centre Iran University of Medical Sciences Tehran Iran; ^16^ Department of Paediatrics and Adolescent Medicine American University of Beirut Beirut Lebanon; ^17^ Department of Obstetrics and Gynaecology, Faculty of Medicine University of Cyberjaya Cyberjaya Malaysia; ^18^ Department of Obstetrics & Gynaecology Hospital Sultanah Aminah, Ministry of Health Johor Bahru Malaysia; ^19^ Directorate of Health Information, Ministry of Health Mexico City Mexico; ^20^ Perined Utrecht The Netherlands; ^21^ Centro de Investigación en Salud Materna e Infantil, Centro de Investigación para el Desarrollo Integral y Sostenible and School of Medicine Universidad Peruana Cayetano Heredia Lima Peru; ^22^ The Generation R Study Group, Department of Paediatrics, Division of Neonatology, Erasmus MC – Sophia Children's Hospital University Medical Centre Rotterdam Rotterdam The Netherlands; ^23^ Hamad Medical Corporation Doha Qatar; ^24^ Department of Obstetrics and Gynaecology Korea University College of Medicine Seoul South Korea; ^25^ Clinical Epidemiology Division, Department of Medicine Solna Karolinska Institutet Stockholm Sweden; ^26^ Department of Health Sciences, College of Life Sciences University of Leicester Leicester UK; ^27^ National Perinatal Epidemiology Unit, Nuffield Department of Population Health University of Oxford Oxford UK; ^28^ School of Natural and Built Environment Queen's University Belfast Belfast UK; ^29^ Queen's Management School Queen's University Belfast Belfast UK; ^30^ Public Health Scotland Edinburgh UK; ^31^ Usher Institute University of Edinburgh Edinburgh UK; ^32^ Department of Maternity and Sexual Health Team Public Health Scotland Edinburgh UK; ^33^ Catholic University of the Maule Región del Maule Chile; ^34^ Department of Wellness and Health Catholic University of Uruguay Montevideo Uruguay

**Keywords:** low birthweight, newborn, preterm birth, size for gestational age

## Abstract

**Objective:**

To examine the prevalence of novel newborn types among 165 million live births in 23 countries from 2000 to 2021.

**Design:**

Population‐based, multi‐country analysis.

**Setting:**

National data systems in 23 middle‐ and high‐income countries.

**Population:**

Liveborn infants.

**Methods:**

Country teams with high‐quality data were invited to be part of the Vulnerable Newborn Measurement Collaboration. We classified live births by six newborn types based on gestational age information (preterm <37 weeks versus term ≥37 weeks) and size for gestational age defined as small (SGA, <10th centile), appropriate (10th–90th centiles), or large (LGA, >90th centile) for gestational age, according to INTERGROWTH‐21st standards. We considered small newborn types of any combination of preterm or SGA, and term + LGA was considered large. Time trends were analysed using 3‐year moving averages for small and large types.

**Main outcome measures:**

Prevalence of six newborn types.

**Results:**

We analysed 165 017 419 live births and the median prevalence of small types was 11.7% – highest in Malaysia (26%) and Qatar (15.7%). Overall, 18.1% of newborns were large (term + LGA) and was highest in Estonia 28.8% and Denmark 25.9%. Time trends of small and large infants were relatively stable in most countries.

**Conclusions:**

The distribution of newborn types varies across the 23 middle‐ and high‐income countries. Small newborn types were highest in west Asian countries and large types were highest in Europe. To better understand the global patterns of these novel newborn types, more information is needed, especially from low‐ and middle‐income countries.

## 1 INTRODUCTION

Newborns who are low birthweight (LBW, <2500 g) have an increased risk of short‐term complications including death during the first 28 days after birth (neonatal mortality) and long‐term adverse health outcomes.^1^, ^2^, ^3^, ^4^, ^5^, ^6^, ^7^, ^8^, ^9^ Around 80% of the world's 2.4 million neonatal deaths are among LBW infants.^10^ Although used for more than a century as an indicator of a suboptimal birth outcome, LBW can arise as the result of preterm birth (‘born too soon’) and small‐for‐gestational age (SGA) (<10th centile for gestational age and sex; ‘born too small’) or both. Reduction in LBW prevalence has been a global target since 1990, but very little progress has been achieved to date.^11^

Globally, an estimated 20.5 million babies were born LBW in 2015,^11^ and 14.8 million babies were born preterm in 2014.^12^ LBW and preterm birth have been estimated at the global, regional and national levels as separate indicators, omitting the important contribution of SGA. This dichotomous approach does not consider the overlap of adverse conditions or the well‐recognised risk for large for gestational age (LGA, >90th centile for gestational age). The prevalence of LGA is increasing globally with the increasing prevalence of women who are overweight and obese, which has been associated with an increase in birth trauma, hospitalisation and long‐term health complications.^4^, ^5^, ^13^, ^14^, ^15^

Using information on gestational age (being born preterm [PT] versus term [T]), size for gestational age, (SGA, LGA or appropriate for gestational age [AGA]), and birthweight (LBW vs nonLBW), we can obtain a set of ten mutually exclusive newborn types. However, given that LBW is caused by preterm and/or SGA, a simplified set of six types (excluding birthweight) could be a practical alternative to describe pathways and patterns of vulnerability and subsequent risks of complications.

A recent *Lancet* commentary called for a better description of the prevalence and mortality risk of newborn types to delineate vulnerability.^16^ This level of granularity could identify babies at the highest risk of complications, help better understand biological mechanisms, inform more targeted and innovative interventions, and accelerate progress towards global LBW and neonatal mortality reduction targets.^17^, ^18^, ^19^ The Vulnerable Newborn Measurement Collaboration is a multi‐country partnership that aims to close this measurement gap by applying standard definitions to classify newborn types and estimate prevalence, mortality risks and mortality attributable to these newborn types.

This paper aims to calculate the prevalence of six newborn types among live births using individual‐level national data and to describe time trends for these types (Table 1).

**TABLE 1:** Key findings.

1. What was known?
Babies born preterm (<37 weeks gestation), low birthweight (<2500 g), or small‐ or large‐for gestational‐age (SGA < 10th centile and LGA > 90th centile) are at higher risk of early mortality and adverse health outcomes later in life. Prevalences of LBW, preterm birth, and SGA/LGA have historically been analysed separately, even though babies may be classified in more than one of these categories, e.g., preterm and SGA
2. What was done that is new?
We have analysed prevalences of novel newborn types in high and middle‐income countries using information on gestational age to classify preterm (PT) vs. term (T) and information on birthweight, gestational age, and newborn sex to classify size‐for‐gestational age as small (SGA), appropriate (AGA), or large (LGA). We identified 23 national datasets with individual‐level information on infants born between 2000 and 2021 and described six newborn categories: four small (PT + SGA, PT + AGA, PT + LGA, T + SGA), one large (T + LGA), and one reference category (T+AGA).
3. What was found?
Our large, pooled dataset of 165 million livebirths provides the first published multi‐country prevalence estimates of vulnerable newborn types. The median prevalence of small types was 11.7%, and was highest in Malaysia (26%) and Qatar (15.7%). Large (T + LGA) types were more common (median prevalence: 18.1%), and comprised over a quarter of all births in Estonia and Denmark. The proportion of small and large newborn types was relatively stable in most countries for the period 2000–2021; however, we observed some increasing time trends of small newborn types in Lebanon and decreasing time trends of large newborn type (T + LGA) in Denmark, Finland, and Sweden. These time trends could be related to shifts and improvements in data systems, data capture, and changes in healthcare such as increases in caesarean section rates.
4. What next?
*Improving the identification of vulnerable newborns in programmes*: These six newborn types could be used in clinical practice and population health to identify subgroups of most vulnerable newborns such as those born preterm and SGA. Compared with the classification of a single indicator of preterm birth or size‐for‐gestational age, the classification of newborns based on a combination of preterm birth and size‐for‐gestational age to define vulnerable newborn types can provide more granular information on baby´s health for targeted interventions, and for tracking changes.
*Research gaps*: More work is needed to assess the prevalence of vulnerable newborn types in regions with the highest burden of small babies such as Sub‐Saharan Africa and Southern Asia. Improvements are also needed in the ascertainment and reporting of reliable gestational age information to enable classification of newborns according to newborn types everywhere.

## 2 METHODS

We analysed routinely collected data on 165 million live births from 1 January 2000 to 31 December 2021 from 23 participating countries. We followed the guidelines for Reporting of Studies conducted using Observational Routinely collected Data (RECORD)^20^ (Appendix S1). Details on ethics approval for all 23 participating countries are in Appendix S2 and all relevant definitions used are provided in Appendix S3.

### 2.1 Data collection and management

An open call for participating countries was widely disseminated through a *Lancet* commentary,^16^ social media and personal emails to researchers who have previously conducted perinatal studies using national routinely collected data. Potential collaborators with population‐level data were invited to complete details in an online survey from August to October 2021.

Collaborators from countries with strong data systems for births defined as having high‐quality criteria based on previous United Nations (UN) preterm birth^12^, ^21^ and LBW^11^ estimates, and with national individual‐level data sets with information on birthweight and gestational age were invited to be part of the Vulnerable Newborn Measurement Collaborative Group: an equitable partnership facilitated by the London School of Hygiene & Tropical Medicine (LSHTM) and the Johns Hopkins University (JHU).

Individual‐level data that met prespecified quality criteria and were collected from the year 2000 in participating countries were included. Quality criteria were defined as: (1) relatively high coverage with at least 80% of live births included in the data set according to the UN reference population, (2) at least 80% of births occurring in healthcare facilities and (3) at least 80% completeness overall for birthweight, gestational age and newborn sex.^22^ For analytical purposes, the three devolved nations from the UK were presented separately, i.e. England & Wales, Northern Ireland and Scotland.

Additional data quality assessment included quantifying the percentage of records for each country‐year by reviewing the median and interquartile range (IQR) of birthweight and gestational age, and detecting evidence of heaping.^23^ Birthweight heaping index was quantified as the number of live births reported at exactly 2500 g divided by the number of live births with reported birthweight that was 249 g below (i.e. 2250–2499 g) and 249 g above (i.e. 2501–2750 g)^24^, ^25^ with low values indicating better reporting practices and lower probability of birthweight heaping. The distribution of birthweight and gestational age was assessed visually and by quantifying the proportion of live births with birthweight below 500 g, below 1000 g or gestational age up to and including 28^+6^ weeks of gestation.

Individual live‐birth records missing birthweight, gestational age or newborn sex were excluded because the size for gestational age could not be calculated. Live‐birth records with implausible values on birthweight (<250 g or ≥6500 g), gestational age (<22^+0^ weeks or >44^+6^ weeks) or an implausible combination of birthweight and gestational age (defined as birthweight ±5 standard deviations from the mean birthweight at each completed week of gestational age) were also excluded. We described the frequency of key baseline characteristics of the women (age, educational attainment, parity) and newborn babies (sex, multiplicity, mode of delivery). Finally, we assessed the proportion of records with insufficient information to categorise them into vulnerable newborn types.

### 2.2 Prevalence of newborn types

Each national team used their data to categorise each included live birth based on gestational age (preterm birth, up to 36^+6^ weeks [PT] or term, 37^+0^ weeks and above [T]), size for gestational age (SGA, AGA, LGA) using the INTERGROWTH‐21st international newborn size for age and sex standards (extended to include newborns from 22^+0^ to 44^+6^)^26^, ^27^, ^28^ (Appendix S4a), and birthweight (LBW <2500 g or nonLBW ≥2500 g).

**FIGURE 1:**
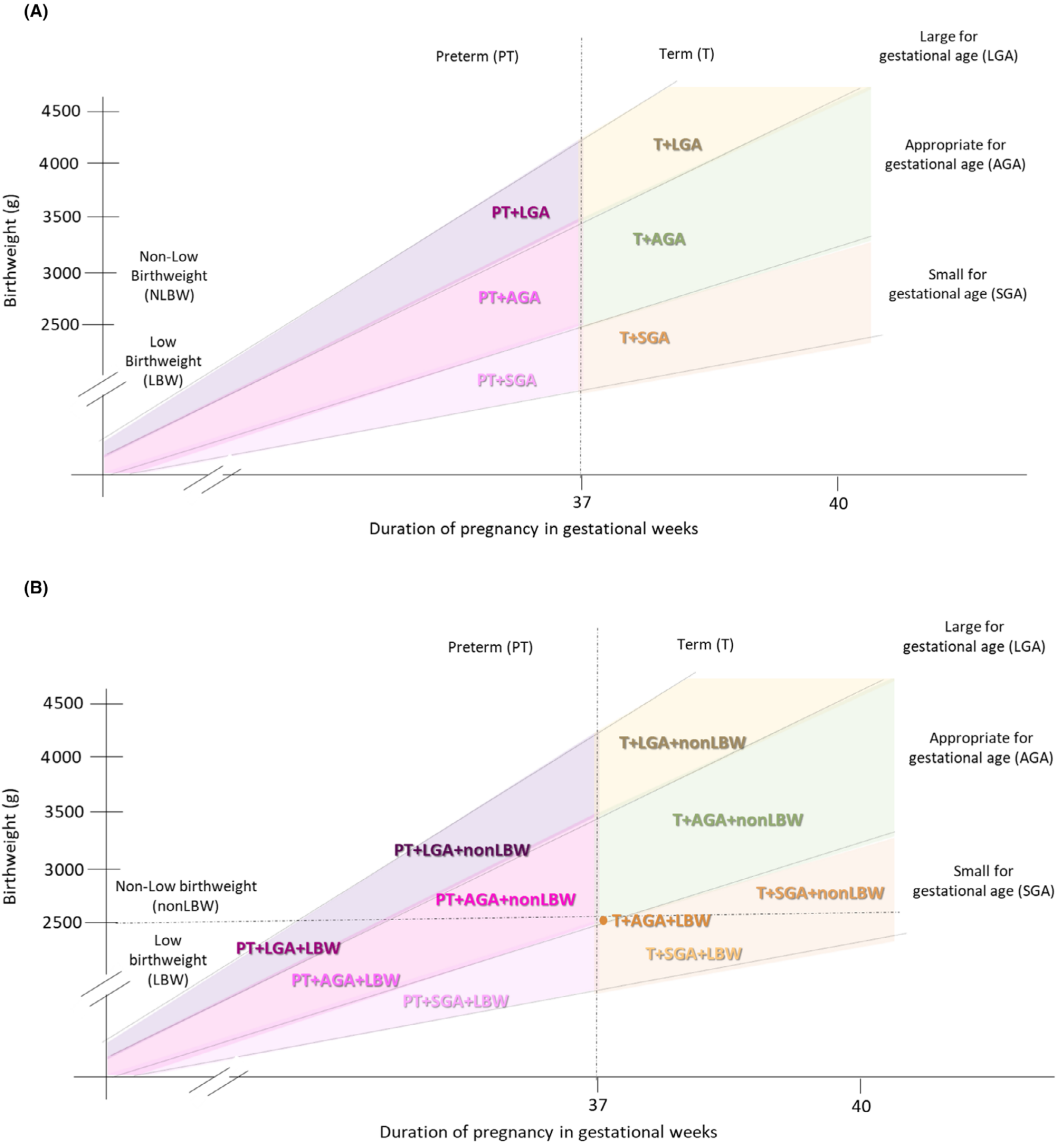
Overview of vulnerable newborn types based on gestational age, size for gestational age and birthweight. This figure illustrates the six newborn types (A) used in the main analysis and more granular expansion of these types adding the birthweight dimension (B) used in the secondary analysis in this paper. Original newborn types proposed by Ashorn et al. are shown as Appendix S4b.

Using gestational age (PT/T) and size for gestational age (SGA/AGA/LGA), we constructed a mutually exclusive set of six newborn types including four small groups (PT + SGA, PT + AGA, PT + LGA, T + SGA), one large group (T + LGA) and one reference group (T + AGA; Figure 1 and Appendix S4b). We defined vulnerable newborn types as ‘small’ (PT and/or SGA) or ‘large’ (T + LGA).

We also performed a secondary analysis with ten categories using all three variables: gestational age (PT/T), size for gestational age (SGA/AGA/LGA) and birthweight (LBW/nonLBW). The ten category types included: eight small groups (T + AGA + LBW, T + SGA + nonLBW, T + SGA + LBW, PT + LGA + nonLBW, PT + LGA + LBW, PT + AGA + nonLBW, PT + AGA + LBW, PT + SGA + LBW), one large group (T + LGA + nonLBW) and one reference group (T + AGA + nonLBW; Figure 1, Appendix S4b).

All analyses were undertaken by the national collaborating teams using standard code in stata (StataCorp, College Station, TX, USA), SAS (SAS Institute, Cary, NC, USA) or R programming languages, co‐developed with the LSHTM/JHU team, to undertake data set cleaning and output results in a standard format. Aggregate national data tables were shared with the coordinating team at LSHTM and pooled into a single data set for further analysis. We performed a sensitivity analysis in countries where missing values for birthweight, sex or gestational age were reported as more than 20% for any country‐year. We compared the prevalence of each newborn type with a subgroup excluding country‐years with more than 20% missingness.

### 2.3 Temporal trends

Temporal changes in the proportion of vulnerable newborn types were estimated for countries reporting data for at least four country‐years. We used 3‐year moving averages to obtain smoothed trends and uncertainty bounds of the calculated annual percentages for small and large vulnerable newborn groups. We defined 3‐year moving average changes of more than 0.5% to denote changes in trend for a particular country‐year. We described changes over two periods of time (from 2000 to 2009 and from 2010 to 2021) to identify changes over two decades.

## 3 RESULTS

**FIGURE 2:**
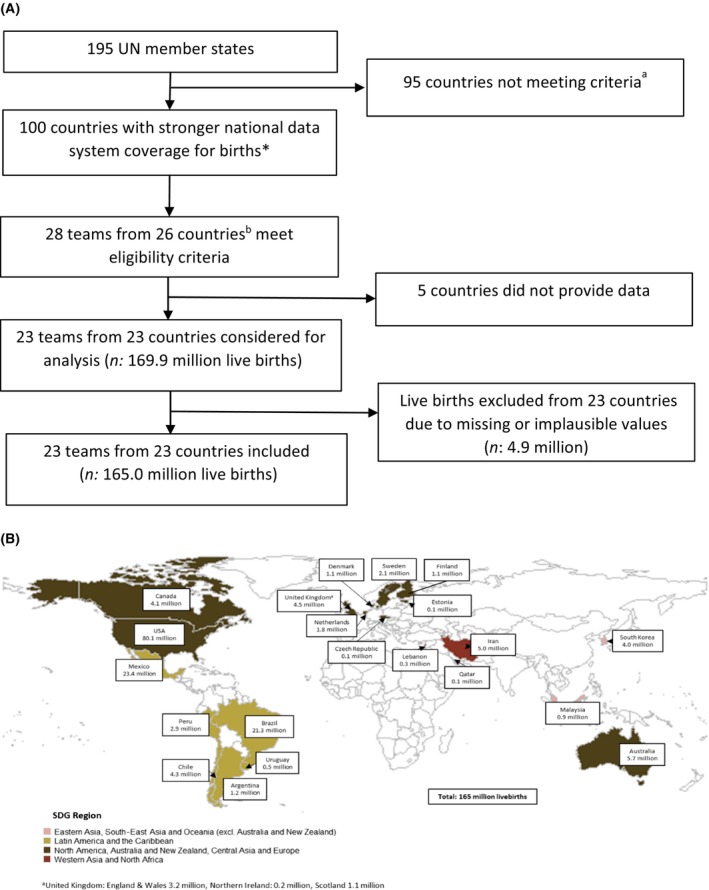
Input data set for Vulnerable Newborn national prevalence. (A) Flow chart for data inclusions and exclusions. ^a^Focus of searches for subnational data see ref. 49 for further details. ^b^Defined as having input data meeting high‐quality criteria for previous UN preterm birth^12^, ^21^ and low birthweight estimates,^11^ or the country having a strong national health system defined as at least 80% facility births. (B) Distribution of 165 million live births included from national data sets in 23 countries, by Sustainable Development Goal (SDG) Region.

Individual‐level data on 169 906 956 live births collected from 23 countries between 2000 and 2021 were considered for analysis. Of the 169 906 956 total live births identified, 4 889 537 (2.9%) records were excluded (Figure 2, Appendix S5a). The most common reason for exclusion was the lack of reporting of gestational age (*n* = 2 752 082; 1.6% of the total records) and this percentage was highest in Malaysia (16.9%) and Brazil (8.7%). Overall, 1 605 371 (0.9%) of records were excluded because of missing birthweight, with Mexico (5.8%) and Lebanon (3.9%) reporting the highest percentage of missingness. Fewer than 0.3% of records were excluded for missing both gestational age and birthweight in the same registry, missing information on sex, implausible birthweight, gestational age records or combinations of birthweight/gestational age. The final data set consisted of 165 017 419 records (97.1% of identified live births). The SDG region of North America, Australia, New Zealand, Central Asia and Europe contributed with 100.9 (61.2%) million live births, followed by Latin America and the Caribbean with 53.7 million (32.6%), western Asia and North Africa contributed 5.4 million (3.3%) and eastern Asia, South‐East Asia, and Oceania had 4.9 (3.0%) million live births. No data were available from southern Asia and Sub‐Saharan Africa (Figure 2).

There were some notable differences in maternal characteristics by region, for instance, there were higher rates of adolescent mothers in Mexico (18.6%) and Brazil (16.3%), and higher rates of caesarean deliveries in Brazil (50.9%), Mexico (42.8%) and Uruguay (42.8%) compared with countries in other regions. Births outside healthcare facilities were more common in the Netherlands where around one‐quarter of births were supervised by a primary care midwife as outpatients in the hospital or at home (26.7%; Table S5b).

More than 97% of birth records had sufficient information to enable classification into vulnerable newborn types (Table 2). In terms of data quality, countries reported a plausible median of birthweight and gestational age for each country‐year of observation with overall proportions of babies below 1000 g (0.6%; range: 0.2–0.8%), and proportion up to and including 28^+6^ weeks of gestation (0.5%; range: 0.3–0.7%; Table 2). The proportion of the smallest babies (<500 g) was 0.1% (range: <0.1% in most countries to 0.3% in Malaysia). There was some evidence of birthweight heaping at 2500 g (3.1%), with the greatest heaping observed in Malaysia (12.9%) and the lowest in Sweden (<0.01%; Table 2).

**TABLE 2:** Quality assessment of data set for Vulnerable Newborn national prevalence in 23 countries, by Sustainable Development Goal (SDG) Region, 2000–2021.

SDG regions and countries	Time period	Total live births	Extreme preterm/low birthweight			Missing values			Implausible combinations	Heaping index
			<500 g	<1000 g	<28 weeks	Birthweight	Gestational age	Sex		
	Range	*n*	%	%	%	%	%	%	%	%
Eastern, South‐East Asia and Oceania	2010–2019	5 212 430	0.2	0.5	0.5	0.3	3.7	0.1	0.0	7.5
South Korea	2010–2019	4 129 808	0.2	0.5	0.5	0.2	0.3	0.0	0.0	3.2
Malaysia	2010–2017	1 082 622	0.3	0.7	0.4	0.8	16.9	0.3	0.2	12.9
Latin America and the Caribbean	2000–2019	57 424 947	0.1	0.4	0.4	2.6	3.7	0.1	0.0	3.6
Argentina	2017–2018	1 266 484	<0.1	0.5	0.4	0.6	1.0	0.8	<0.1	5.5
Brazil	2011–2018	23 439 789	0.1	0.6	0.5	0.1	8.7	<0.1	0.1	2.3
Chile	2000–2017	4 330 675	0.1	0.4	0.4	0.2	0.0	<0.1	<0.1	2.6
Mexico	2008–2019	24 955 172	<0.1	0.2	0.3	5.8	0.4	0.1	<0.1	6.4
Peru	2012–2019	2 933 482	<0.1	0.3	0.3	<0.1	<0.1	0.0	<0.1	3.6
Uruguay	2009–2020	499 345	<0.1	0.4	0.4	0.1	1.1	0.0	<0.1	2.8
North America, Australia, New Zealand, Central Asia and Europe	2000–2021	101 537 859	0.1	0.6	0.6	0.1	0.4	<0.1	<0.1	1.4
Australia	2000–2019	5 755 869	0.1	0.4	0.4	<0.1	<0.1	<0.1	<0.1	0.3
Canada^a^	2005–2019	4 163 541	0.1	0.5	0.5	<0.1	0.8	<0.1	<0.1	1.0
Czech Republic	2019	112 231	<0.1	0.3	0.3	1.3	2.4	0.0	<0.1	1.8
Denmark	2000–2017	1 125 560	0.1	0.4	0.4	1.9	1.7	<0.1	<0.1	3.0
England & Wales	2015–2019	3 212 492	0.1	0.5	0.5	0.0	0.0	0.0	0.0	2.3
Estonia	2015–2020	82 427	<0.1	0.3	0.4	0.0	0.0	0.0	0.0	0.9
Finland	2000–2019	1 127 344	<0.1	0.3	0.3	<0.1	<0.1	0.0	<0.1	2.4
The Netherlands	2010–2020	1 861 400	<0.1	0.4	0.4	0.1	0.7	<0.1	<0.1	1.4
Northern Ireland	2016–2021	155 992	0.1	0.4	0.4	0.1	<0.1	<0.1	<0.1	2.8
Scotland	2000–2020	1 127 984	<0.1	0.4	0.4	0.1	0.1	<0.1	<0.1	2.7
Sweden	2000–2019	2 102 671	0.0	0.0	0.0	0.0	0.0	0.0	0.0	0.0
USA	2000–2019	80 710 348	0.2	0.7	0.7	0.1	0.4	<0.1	<0.1	0.5
Western Asia and North Africa	2001–2021	5 731 720	0.2	0.8	0.7	0.3	0.3	0.3	0.1	9.2
Iran	2017–2021	5 308 356	0.2	0.8	0.7	<0.1	<0.1	<0.1	0.1	11.6
Lebanon	2001–2019	327 458	<0.1	0.3	0.3	3.9	4.8	4.3	<0.1	10.2
Qatar^b^	2016–2019	95 906	<0.1	0.5	0.6	0.4	1.3	<0.1	<0.1	1.6

^a^ Excluding Quebec.

^b^ Excluding births with missing birth status (live birth or stillbirth).

### 3.1 Prevalence of newborn types

Preterm and SGA median proportions were higher in eastern and southern Asia (preterm 9.9%, SGA 11%), followed by western Asia (preterm 9.6%, SGA 6.6%) and Latin America and the Caribbean (preterm 8.2%, SGA 5.4%). Malaysia reported the highest prevalence of preterm (11.8%) and SGA (15.7%) babies. In contrast, LGA infants were more common in North America, Australia, Central Asia, and Europe (median 22.9%) ranging from 15% in the Czech Republic to 29.7% in Estonia (Appendix S6a).

**FIGURE 3:**
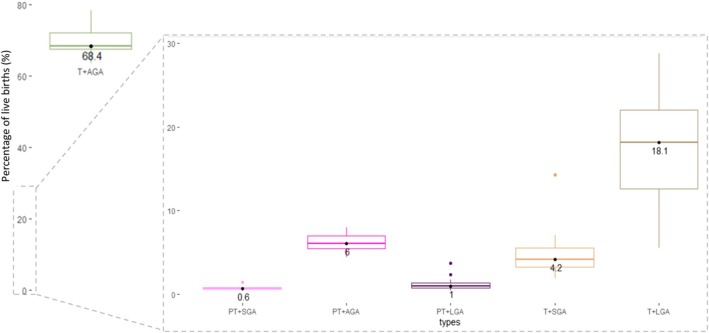
Median and interquartile range of national prevalence of six newborn types among 165 017 419 live births included from 23 countries. National data are presented as separate analyses as provided by devolved nations (England & Wales, Northern Ireland, Scotland) from the United Kingdom.

The median prevalence of any type of small newborn type (preterm or SGA) was 11.7% (IQR 9.9–14.2%) ranging from 7.6% in Estonia (IQR 7.6–7.7%) to 26% (25.7–26.3%) in Malaysia. Among small babies, preterm types were: 6% for PT + AGA, 1% for PT + LGA and 0.6% for PT + SGA. The prevalence of small babies at term (T + SGA) was 4.2% (Table 3 and Table S6b). The median prevalence for large babies (T + LGA) was 18.1% (IQR 12.6–22.1%) ranging from 5.6% (IQR 5.5–5.7%) in Malaysia to more than one‐quarter (28.8%, IQR 28.3–29.4%) in Estonia. In all countries, most live births were T + AGA (median 68.4%, IQR 67.5–72.1%; Figure 3, Table S6b,c).

**TABLE 3:** Median and interquartile range of national prevalences of six newborn types and small types among 165 017 419 live births, overall and by Sustainable Development Goal Region.

Variables	Six newborn types						Small types^a^
	PT + SGA	PT + AGA	PT + LGA	T + SGA	T + AGA	T + LGA	
	Median	Median	Median	Median	Median	Median	Median
	IQR	IQR	IQR	IQR	IQR	IQR	IQR
Overall^b^	0.6	6.0	1.0	4.2	68.4	18.1	11.7
	(0.6–0.8)	(5.4–7.0)	(0.8–1.4)	(4.2–3.3)	(67.5–72.1)	(12.6–22.1)	(9.9–14.2)
Eastern, South‐East Asia and Oceania	1.0	7.3	1.5	9.9	73.5	6.7	19.8
	(0.8–1.2)	(7.0–7.7)	(1.1–1.9)	(7.8–12.1)	(71.0–76.0)	(6.1–7.3)	(16.7–22.9)
Latin America and the Caribbean	0.7	6.3	1.0	4.6	69.6	16.5	12.9
	(0.6–0.8)	(5.4–7.0)	(0.8–1.6)	(4.0–6.0)	(69.0–72.7)	(13.2–17.8)	(11.6–14.0)
North America, Australia/New Zealand, Central Asia and Europe	0.6	5.7	0.9	3.3	67.5	22.1	10.5
	(0.6–0.7)	(5.1–6.1)	(0.7–1.0)	(3.0–4.1)	(66.6–67.9)	(19.9–24.0)	(9.5–11.7)
Western Asia and North Africa	0.9	7.2	1.4	5.7	72.7	11.9	15.4
	(0.8–0.9)	(6.9–7.5)	(1.4–1.5)	(5.6–5.7)	(72.1–73.2)	(11.9–12.3)	(14.8–15.5)

^a^ Small types include: PT + SGA, PT + AGA, PT + LGA, T + SGA.

^b^ Twenty‐three national data sets.

**FIGURE 4:**
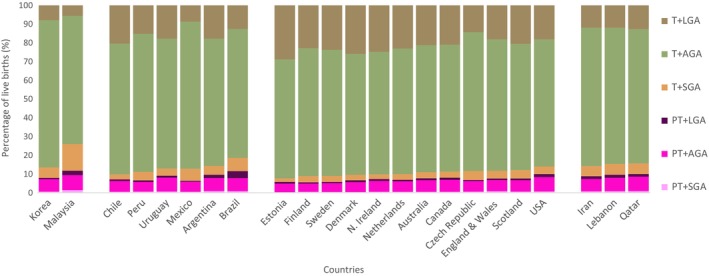
Prevalence of six newborn types among 165 017 419 live births included from 23 countries, by country and Sustainable Development Goal Region. National data are presented as separate data as provided by devolved nations (England & Wales, Northern Ireland, Scotland) from the United Kingdom.

Regional variation was noted, with small vulnerable newborn types more common in western Asia (Malaysia 26.9%, Qatar 15.7%), Latin America (Brazil 18.6%, Argentina 14.3%) and North America and Europe (USA 14.1%, Scotland 12.1%; Figure 4). We found that large (LGA + T) babies were more common in European countries (e.g. Estonia 28.8%, Denmark 25.9%, Northern Ireland 24.8%), Australia (21.2%), Canada (21.0%) and in some countries in Latin America, including Chile (20.4%) and Argentina (17.8%; Figure 4, Table S6b,c).

Using the classification of ten newborn types, four types were split into two smaller groups (LBW and nonLBW). The group PT + AGA (median 6%) included PT + AGA + LBW (median 3.6%) and PT + AGA + nonLBW (median 2.3%). The group PT + LGA (median 1%) included those PT + LGA + LBW (median 0.2%) and PT + LGA + nonLBW (median 0.8%). Of the term infants, the group T + SGA (median 4.2%) comprised babies that were T + SGA + LBW (median 1.9%) and T + SGA + nonLBW (median 2.3%). The reference group T + AGA (median 68.4%) included mainly nonLBW babies (T + AGA + nonLBW, median 68.1%) and a very small group of LBW babies (T + AGA + LBW, median 0.5%; Appendix S6d).

In the sensitivity analyses ten individual country‐years with more than 20% missing data were excluded; as such the proportions of six newborn types were reassessed in two countries, Malaysia (3 years) and Brazil (1 year). The sensitivity analyses showed similar results to the main analyses concerning the overall prevalence for each type in both Malaysia and Brazil (Appendix S6e).

### 3.2 Temporal trends

**FIGURE 5:**
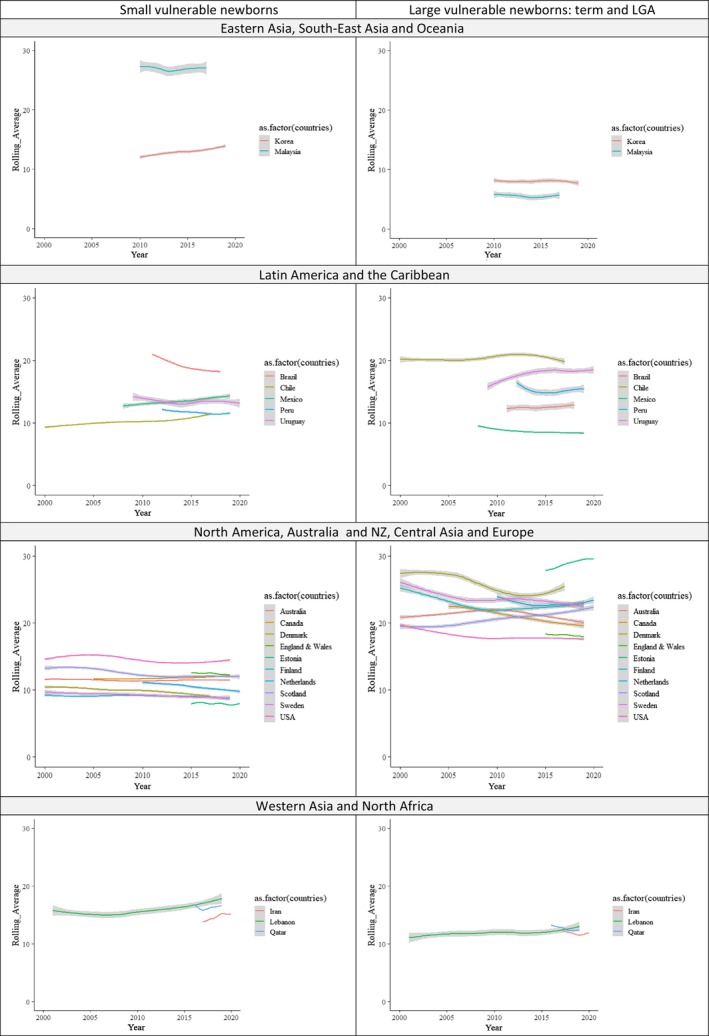
Rolling average of the percentage of live births of different newborn types in 20 countries. Excluding Argentina, the Czech Republic and Northern Ireland for providing pooled data or less than three disaggregated years. Small vulnerable newborns include: PT + SGA, PT + AGA, PT + LGA, T + SGA. Coloured lines are Loess smoothers, grey shading shows the confidence intervals.

Time trends in the prevalence of newborn types were estimated for 20 of the 23 countries with at least four individual years of data (Figure 5). Two national data sets were excluded as they provided data of only two individual years (Argentina and Czech Republic) and one as it provided aggregated data (Northern Ireland). In general, data availability increased with calendar years as more countries contributed data between 2010 and 2021 (19 countries and 173 country‐years) compared with the period from 2000 to 2009 (ten countries and 87 country‐years; Table S6f).

Small vulnerable newborn types appeared to decrease over time in Brazil (20.3% in 2012, 19.7% in 2013 and 19.1% in 2014) and fluctuate in Lebanon (from 15.5% in 2010 to 16.1% in 2011 and from 15.4% in 2015 to 17.6% in 2016). Reduction in the proportion of large newborns was noted in Denmark (27.1% in 2006, 26.4% in 2007 and 25.7% in 2008), Finland (24.1% in 2004, 23.5% in 2005 and 22.8% in 2006) and Sweden (24.5% in 2005 to 23.3% in 2006), whereas this proportion increased in Uruguay during the second decade (16.7% in 2011 to 17.7% in 2012).

## 4 DISCUSSION

### 4.1 Main findings

We have, for the first time, analysed a multi‐country national data set of 165 million live births from 23 countries according to vulnerable newborn types. We found that babies with at least one condition for being small were more likely to be born in western Asian (Middle Eastern) and Latin American countries (Malaysia 26% of all live births, Qatar 15.7%, Lebanon 15.4%, Brazil 18.6%, Argentina 14.3% and Mexico 12.9%). However, even in the high‐income settings of North America and Europe, which generally had lower rates of small vulnerable newborn types, had relatively high rates (USA 14.1%, Scotland 12.1%). In contrast, large (LGA + T) babies were more common in Europe (e.g. Estonia 28.8%, Denmark 25.9%, Northern Ireland 24.8%), Australia (21.2%), Canada (21.0%) and Chile (20.4%).

Time trends in vulnerable newborn types over the last two decades were assessed in 20 countries. We found that most of the included countries did not experience reductions in rates of small newborn types, supporting the evidence from other data sources and global estimates on the lack of progress in reducing LBW and preterm birth.^11^, ^21^ Minor fluctuations in small babies were noted in some countries, which could be explained partially by temporal improvements in the identification of preterm babies in routine systems and improvements in data capture, which are country‐specific.^21^ For instance, the increase in small types in Lebanon can be explained by the influx of Syrian refugees who are at particular risk of suffering maternal and neonatal adverse outcomes^29^, ^30^, ^31^ and the increased reporting of preterm babies (<28^+0^ weeks) from 0.2% in 2012 to 0.3% in 2019. We found reductions in T+LGA in Denmark, Finland and Sweden during the period 2000 to 2009, this effect can be related to delivery practices, screening and control of gestational diabetes.^32^, ^33^, ^34^

### 4.2 Interpretation

Low birthweight has been a widely used marker of newborn vulnerability associated with an elevated risk of mortality and chronic health conditions.^11^, ^17^, ^35^ The use of this threshold (<2500 g) offers some advantages in identifying at‐risk newborns in settings where gestational age assessment is not accurate and underlying reasons for LBW cannot be identified.^11^ Delineating short gestation (preterm) and potentially suboptimal intrauterine growth (SGA as a proxy) is key to informing preventive programmes and policies in all settings to implement targeted public health interventions during pregnancy and the perinatal period. Though the prevalence of babies born both preterm and SGA is low (median 0.6%, IQR 0.6–0.8%), these infants are at higher risk of mortality^36^ and chronic diseases later in life compared with those born either preterm only or SGA only.^17^, ^37^, ^38^ There is inadequate research on the impacts of being born LGA. LGA babies born preterm may differ from those born at term and the vulnerable newborn types classification helps to identify particular determinants and further examine the outcomes of these babies.

Using six newborn type categories rather than ten is a more parsimonious approach and the reduced number of categories may be easier to implement in routine practice while still identifying newborn babies with relatively homogeneous characteristics and consequent premature mortality risks.^36^, ^39^, ^40^, ^41^

Preterm birth is a strong predictor of mortality and longer‐term adverse neurodevelopmental outcomes, but these risks differ depending on gestational age (e.g. extremely preterm versus late preterm). Future work should examine several preterm categories based on maturity, e.g. extremely preterm, very preterm and moderate or late preterm, and further classifications allowing differentiation between spontaneous and clinician‐initiated births.^42^

### 4.3 Strengths and limitations

This novel analysis of newborn types has several strengths, notably the large study size including 165 million live births collected in 23 countries via routine national data systems with high coverage of the national newborn populations. The national data sets had a high level of completeness for the key variables of birthweight, gestational age and sex, and covered a wide period from 2000 to 2021 enabling a comprehensive assessment of the prevalence of these types in different national populations over time. We were also able to extend the original INTERGROWTH‐21st models that initially covered the period from 24^+0^ to 42^+6^ weeks by extrapolating the range of gestational age in very preterm and post‐term babies enabling consistency with International Classification of Diseases periods of gestational cut‐offs.^43^ The differences in reporting practices for births between countries may impact the comparability between countries (Appendix S5g). For example, the high percentage of all live births that occur before 28 weeks of gestation in the USA (0.7%) may be due to more complete registration of very premature babies associated with the 20‐week threshold for reporting fetal deaths.^44^, ^45^

Gestational age assessment methods are known to vary in accuracy, with methods including first‐trimester ultrasound measurement being the most accurate, and last menstrual period dating being less accurate and leading to overestimation of the proportion of preterm babies.^46^ Most data sets from high‐income settings with widespread coverage of early ultrasound assessments use the best obstetric estimates. The method of gestational age assessment was not stated in data sets collected in Asia, North Africa and Latin America. If last menstrual period was the predominant method used in these countries, this might have led to some misclassification and impacted the comparability between countries. In addition, although this analysis included only live births, stillbirths are an important part of the overall assessment of adverse birth outcomes accounting for 2 million deaths annually,^47^, ^48^ so they are the focus of another paper in this supplement.^39^

Sub‐Saharan Africa and southern Asia account for about 70% of the world's births and a disproportionate burden of adverse birth outcomes, notably stillbirths and neonatal deaths. Despite extensive attempts, we did not identify countries meeting the inclusion criteria from these regions to join our collaborative project. To address this gap, our collaboration included data from population‐based research studies from these regions. These research studies were relatively small (a total of 0.5 million live births from 45 studies), and none were designed to be nationally representative, which limits their generalisability. Analyses of these studies suggested a higher prevalence of small newborn types (median 37.6%), and fewer LGA babies (median 3.3%), however, these results were mainly driven by community‐based data in southern Asia.^49^ With over 80% of the world's births occurring in facilities and increasing coverage of routine health management information systems, there are opportunities to improve the collection and use of these data. Unfortunately, most data are available only in an aggregated form and so preclude the identification of specific newborn types, which requires individual‐level data.^50^

## 5 CONCLUSIONS

This study provides the first multi‐country analysis of specific newborn types, based on 165 million live births from 23 high‐ and upper‐middle‐income countries across four Sustainable Development Goal regions. Identification of specific newborn types was highly feasible provided individual‐level data were available. Overall, small vulnerable newborn types were highly prevalent in participating countries, and large newborn types were even more common. These analyses provide a baseline overview of vulnerable newborn types, an important next step is to examine these groups with respect to mortality, which is reported in another paper in this supplement.^40^ Elsewhere we also report regional and global estimates for the six vulnerable newborn type categories.^51^ Longer follow up, and possible electronic cohort analyses would be a useful next step to examine the lifelong impact of each newborn type.

## AUTHOR CONTRIBUTIONS

The Vulnerable Newborn collaborative was planned by JEL and REB. This analysis was designed by HB and EOO with JEL. All authors contributed to the study protocol, with inputs from the wider Vulnerable Newborn Measurement Collaboration. Country data analyses were undertaken and revised by VP, LG, AG, KW, ESP, IRF, SL, QW, FM, RCC, PV, JJ, EHP, HTS, LS, LA, MG, MH, MML, KAY, AAB, SDK, RJ, AB, LB, AEvD, LH, HGQP, KNCT, FA, MAlQ, GJC, HYK, NR, JS, LKS, JK, EL, NR, RW, KM, IP, GP. Pooled analysis was undertaken by LSI with EOO and EB. The manuscript was drafted by LSI and JY with HB, EOO and JEL. JY, YBO and all authors helped revise the manuscript. All authors reviewed and agreed on the final version.

## CONFLICT OF INTEREST STATEMENT

None declared.

## FUNDING INFORMATION

Children's Investment Fund Foundation, prime grant 1803‐02535. The funders had no role in the study design, data collection, analysis or interpretation of the paper.

## AUSTRALIAN DATA DISCLOSURES

We are grateful to Consultative Council on Obstetric and Paediatric Mortality and Morbidity (CCOPMM) for providing access to the data used for this project and for the assistance of the staff at Safer Care Victoria. The conclusions, findings, opinions and views or recommendations expressed in this paper are strictly those of the author(s). They do not necessarily reflect those of CCOPMM. We would like to acknowledge and thank the NT Perinatal Data team for access to the Northern Territory perinatal data collection. Australian data were provided to the CRE team and international team with small numbers, those less than 5, suppressed.

## ETHICS APPROVAL

The Vulnerable Newborn Measurement Collaboration was granted ethical approval from the Institutional Review Boards of the London School of Hygiene & Tropical Medicine (ref: 22858, date of approval: 17 May 2021) and Johns Hopkins Bloomberg School of Public Health (IRB No: 16439, date of approval 8 May 2021). All 23 country teams had ethical approval for use of data or exemptions based on the current remit.

## Supporting information


**Appendix S1–S7.** Supporting Information

## Data Availability

Data sharing and transfer agreements were jointly developed and signed by all collaborating partners. All data used in these analyses are available in the Supplementary Information. The pooled aggregate data will be available at https://doi.org/10.17037/DATA.00003095 at the time of publication with the exception of those from countries where data sharing is not permitted.

## APPENDIX 1

### National Collaborative Group for Vulnerable Newborn Prevalence

Australia: Vicki Flenady; Adrienne Gordon; Kara Warrilow; Harriet Lawford. Argentina: Veronica Pingray; Luz Gibbons; Gabriela Cormick; Jose Belizan, Carlos Guevel. Brazil: Enny S. Paixao; Mauricio Lima Barreto; Ila Rocha Falcão. Canada: Sarka Lisonkova; Qi Wen. Chile: Francisco Mardones; Raúl Caullier‐Cisterna, José Acuña. Czech Republic: Petr Velebil; Jitka Jírová. Denmark: Erzsébet Horváth‐Puhó, Henrik Toft Sørensen. Estonia: Luule Sakkeus; Liili Abuladze. Finland: Mika Gissler. Iran: Mohammad Heidarzadeh; Maziar Moradi‐Lakeh; Narjes Khalili. Lebanon: Khalid A. Yunis; Ayah Al Bizri; Pascale Nakad. Malaysia: Shamala Devi Karalasingam; J. Ravichandran R. Jeganathan; Nurakman Binti Baharum. Mexico: Lorena Suárez‐Idueta; Arturo Barranco Flores; Jesus Felipe Gonzalez Roldan; Sonia Lopez Alvarez. Netherlands: Lisa Broeders; Aimée E. van Dijk. Peru: Hugo G. Quezada‐Pinedo; Kim N. Cajachagua‐Torres; Wilmer Cristobal Guzman‐Vilca; Carla Tarazona‐Meza; Rodrigo M. Carrillo‐Larco; Luis Huicho. Qatar: Fawziya Alyafei; Mai AlQubaisi; Tawa O. Olukade; Hamdy A. Ali; Mohamad Rami Alturk. South Korea: Geum Joon Cho; Ho Yeon Kim. Sweden: Neda Razaz; Jonas Söderling. UK_England and Wales: Lucy K. Smith; Bradley N. Manktelow; Ruth J. Matthews; Elizabeth Draper; Alan Fenton; Jennifer Kurinczuk. UK_Northern Ireland: Estelle Lowry; Neil Rowland. UK_Scotland: Rachael Wood; Celina Davis; Kirsten Monteath; Samantha Clarke. Uruguay: Isabel Pereyra, Gabriella Pravia. USA: Sarka Lisonkova; Qi Wen.

### Vulnerable Newborn Measurement Core Group

LSHTM: Joy E. Lawn; Hannah Blencowe; Eric Ohuma; Yemisrach B. Okwaraji; Judith Yargawa; Ellen Bradley. JHU: Robert E. Black; Joanne Katz; Daniel Erchick; Elizabeth A. Hazel; Michael Diaz; Anne C. C. Lee.
