# Supplementary material for: Vulnerable newborn types: Analysis of population‐based registries for 165 million births in 23 countries, 2000–2021
Source: BJOG. 2023 May 8;132(Suppl 8):S5–S19. doi: 10.1111/1471-0528.17505 (PMC12678069; doi:10.1111/1471-0528.17505)
Supplement: Supplementary file 1 — Appendix S1–S7. Supporting Information [file BJO-132-S5-s001.docx]

**SUPPLEMENT TITLE**: Vulnerable Newborn multi-country analyses related to preterm births or small-for-gestational age

**PAPER TITLE**

Vulnerable newborn types: Analysis of population-based registries for 165 million births in 23 countries, 2000 to 2021

**PAPER RUNNING TITLE**

Vulnerable Newborn Types in 23 countries

**SUPPORTING INFORMATION**

Table of contents

[**S1: RECORD guidelines checklist** 2](#_Toc131071761)

[**S2: Ethics approval or exemptions of Institutional Review Boards** 7](#_Toc131071762)

[**S3: Definitions** 8](#_Toc131071763)

[**S4: Newborn types based on birthweight, gestational age, and size for gestational age according to INTERGROWTH-21st newborn size standards.** 9](#_Toc131071764)

[S4a: Methods used to extend INTERGROWTH-21st newborn size standards from 22^+0^ to 44^+6^ 9](#_Toc131071765)

[S4b: Figure illustrating original newborn types 10](#_Toc131071766)

[**S5: Additional information on input data** 11](#_Toc131071767)

[S5a: Flowchart of database construction. 11](#_Toc131071768)

[S5b: Maternal baseline characteristics of livebirths in 21 participant countries, by SDG Regions 12](#_Toc131071769)

[**S6: Additional analyses and results** 14](#_Toc131071770)

[S6a: Preterm, LBW, SGA, and LGA amongst 165,017,419 livebirths included from 23 countries, by SDG Region 14](#_Toc131071771)

[S6b: Prevalence of six newborn types among livebirths (n: 165,017,419), by SDG Region 15](#_Toc131071772)

[S6c: Six newborn types sorted in ascending order (n: 165,017,419) 16](#_Toc131071773)

[S6d: Ten newborn types amongst livebirths (n=165,017,419), by country 17](#_Toc131071774)

[S6e: Sensitivity analysis for six newborn Types prevalence excluding years with missing gestational age >20%. 19](#_Toc131071775)

[S6f: Three-year rolling average changes for Vulnerable Newborn (VN) Types in 19 countries*, 2000 to 2009 vs 2010 to 2021 20](#_Toc131071776)

[S6g: Reporting practices in participating countries 21](#_Toc131071777)

[**S7. Additional references** 24](#_Toc131071778)

# **S1: RECORD guidelines checklist**

|  | **#** | **STROBE items** | **Location in manuscript where items are reported** | **RECORD items** | **Location in manuscript where items are reported** |
| --- | --- | --- | --- | --- | --- |
| **Title and abstract** | | | | | |
|  | 1 | (a) Indicate the study’s design with a commonly used term in the title or the abstract (b) Provide in the abstract an informative and balanced summary of what was done and what was found |  | RECORD 1.1: The type of data used should be specified in the title or abstract. When possible, the name of the databases used should be included.  RECORD 1.2: If applicable, the geographic region and timeframe within which the study took place should be reported in the title or abstract.  RECORD 1.3: If linkage between databases was conducted for the study, this should be clearly stated in the title or abstract. | Title: “Vulnerable newborn types: Analysis of population-based registries for 165 million births in 23 countries, 2000 to 2021” |
| **Introduction** | | | | | |
| Background rationale | 2 | Explain the scientific background and rationale for the investigation being reported |  |  | Paragraph 1-4 |
| Objectives | 3 | State specific objectives, including any prespecified hypotheses |  |  | Paragraph 5 |
| **Methods** | | | | | |
| Study Design | 4 | Present key elements of study design early in the paper |  |  | Abstract and paragraph 1 of the Methods section. |
| Setting | 5 | Describe the setting, locations, and relevant dates, including periods of recruitment, exposure, follow-up, and data collection |  |  | Abstract and paragraphs 2-3 of the Methods section. |
| Participants | 6 | *(a) Cohort study* - Give the eligibility criteria, and the sources and methods of selection of participants. Describe methods of follow-up  *Case-control study* - Give the eligibility criteria, and the sources and methods of case ascertainment and control selection. Give the rationale for the choice of cases and controls  *Cross-sectional study* - Give the eligibility criteria, and the sources and methods of selection of participants  *(b) Cohort study* - For matched studies, give matching criteria and number of exposed and unexposed  *Case-control study* - For matched studies, give matching criteria and the number of controls per case |  | RECORD 6.1: The methods of study population selection (such as codes or algorithms used to identify subjects) should be listed in detail. If this is not possible, an explanation should be provided.  RECORD 6.2: Any validation studies of the codes or algorithms used to select the population should be referenced. If validation was conducted for this study and not published elsewhere, detailed methods and results should be provided.  RECORD 6.3: If the study involved linkage of databases, consider use of a flow diagram or other graphical display to demonstrate the data linkage process, including the number of individuals with linked data at each stage. | Paragraphs 3-5 of the Methods section |
| Variables | 7 | Clearly define all outcomes, exposures, predictors, potential confounders, and effect modifiers. Give diagnostic criteria, if applicable. |  | RECORD 7.1: A complete list of codes and algorithms used to classify exposures, outcomes, confounders, and effect modifiers should be provided. If these cannot be reported, an explanation should be provided. | Paragraphs 6-7 **(Under the subheading Prevalence of Newborn types)** |
| Data sources/ measurement | 8 | For each variable of interest, give sources of data and details of methods of assessment (measurement).  Describe comparability of assessment methods if there is more than one group |  |  | Paragraph 6-7 **(Under the subheading Prevalence of Newborn types)** |
| Bias | 9 | Describe any efforts to address potential sources of bias |  |  | Paragraph 4  **(Under the subheading Data collection and management)** |
| Study size | 10 | Explain how the study size was arrived at |  |  | Figure 1 |
| Quantitative variables | 11 | Explain how quantitative variables were handled in the analyses. If applicable, describe which groupings were chosen, and why |  |  | Paragraphs 6-7  **(Under the subheading Prevalence of Newborn types)** |
| Statistical methods | 12 | (a) Describe all statistical methods, including those used to control for confounding  (b) Describe any methods used to examine subgroups and interactions  (c) Explain how missing data were addressed  (d) *Cohort study* - If applicable, explain how loss to follow-up was addressed  *Case-control study* - If applicable, explain how matching of cases and controls was addressed  *Cross-sectional study* - If applicable, describe analytical methods taking account of sampling strategy  (e) Describe any sensitivity analyses |  |  | Paragraphs 7-8  **(Under the subheading Prevalence of newbotn types)**  Also, paragraph 9  **(Under the subheading Temporal Trends)** |
| Data access and cleaning methods |  | .. |  | RECORD 12.1: Authors should describe the extent to which the investigators had access to the database population used to create the study population.  RECORD 12.2: Authors should provide information on the data cleaning methods used in the study. | Paragraph 8 in Methods **(Under the subheading Prevalence of newbotn types)** |
| Linkage |  | .. |  | RECORD 12.3: State whether the study included person-level, institutional-level, or other data linkage across two or more databases. The methods of linkage and methods of linkage quality evaluation should be provided. | No linkage required to calculate prevalence and time trends |
| **Results** | | | | | |
| Participants | 13 | (a) Report the numbers of individuals at each stage of the study (*e.g.*, numbers potentially eligible, examined for eligibility, confirmed eligible, included in the study, completing follow-up, and analysed)  (b) Give reasons for non-participation at each stage.  (c) Consider use of a flow diagram |  | RECORD 13.1: Describe in detail the selection of the persons included in the study (*i.e.,* study population selection) including filtering based on data quality, data availability and linkage. The selection of included persons can be described in the text and/or by means of the study flow diagram. | Paragraph 4 of the Result section, and supporting information S5b (flow chart) |
| Descriptive data | 14 | (a) Give characteristics of study participants (*e.g.*, demographic, clinical, social) and information on exposures and potential confounders  (b) Indicate the number of participants with missing data for each variable of interest  (c) *Cohort study* - summarise follow-up time (*e.g.*, average and total amount) |  |  | Clinical baseline characteristics of livebirth records in paragraph 2 of the Result section.  Missing data for each variable of interest in supporting information (Table S5a) |
| Outcome data | 15 | *Cohort study* - Report numbers of outcome events or summary measures over time  *Case-control study* - Report numbers in each exposure category, or summary measures of exposure  *Cross-sectional study* - Report numbers of outcome events or summary measures |  |  | Supporting information S5a |
| Main results | 16 | (a) Give unadjusted estimates and, if applicable, confounder-adjusted estimates and their precision (e.g., 95% confidence interval). Make clear which confounders were adjusted for and why they were included  (b) Report category boundaries when continuous variables were categorized  (c) If relevant, consider translating estimates of relative risk into absolute risk for a meaningful time period |  |  | Unadjusted estimates in paragraph 6-7 in Result **(Under the subheading Prevalence of Vulnerable Newborn Types)** |
| Other analyses | 17 | Report other analyses done—e.g., analyses of subgroups and interactions, and sensitivity analyses |  |  | Sensitivity analysis in paragraph 6 **(Under the subheading Prevalence of Newborn Types)**  Time trends in paragraphs 10-11 in Results **(Under the subheading Time trends)** |
| **Discussion** | | | | | |
| Key results | 18 | Summarise key results with reference to study objectives |  |  | Paragraph 1-2 of the discussion section. |
| Limitations | 19 | Discuss limitations of the study, taking into account sources of potential bias or imprecision. Discuss both direction and magnitude of any potential bias |  | RECORD 19.1: Discuss the implications of using data that were not created or collected to answer the specific research question(s). Include discussion of misclassification bias, unmeasured confounding, missing data, and changing eligibility over time, as they pertain to the study being reported. | Paragraph 7-8 of the discussion **(Under the subheading Strengths and Limitations)** |
| Interpretation | 20 | Give a cautious overall interpretation of results considering objectives, limitations, multiplicity of analyses, results from similar studies, and other relevant evidence |  |  | Paragraph 3-4 in the discussion section **(Under the subheading Implications)** |
| Generalisability | 21 | Discuss the generalisability (external validity) of the study results |  |  | Paragraph under the subheading conclusions |
| **Other Information** | | | | | |
| Funding | 22 | Give the source of funding and the role of the funders for the present study and, if applicable, for the original study on which the present article is based |  |  | We included grant details in Abstract and under the heading Funding role |
| Accessibility of protocol, raw data, and programming code |  | .. |  | RECORD 22.1: Authors should provide information on how to access any supplemental information such as the study protocol, raw data, or programming code. | We included a section on availability of data and material and link |

Source ^1^

# **S2: Ethics approval or exemptions of Institutional Review Boards**

| **Country of origin for data** | **Institutional Review Board(s) or data access provider** | **Ref/Number** | **Date of approval** |
| --- | --- | --- | --- |
|  | LSHTM - Observational / Interventions Research Ethics Committee | 22858 | 17^th^ May 2021 |
| Australia | Australian Institute of Health and Welfare Ethics Committee | EO2018/2/451 | 4^th^ May 2021 |
| Brazil | Federal University of Bahia’s Institute of Public Health Ethics Committee | 18022319.4.0000.5030 | 3^rd^ September 2019 |
| Canada | UBC C&W Research Ethics Board | H21-00653 | 31^st^ March 2021 |
| Estonia | Ethics Committee of National Institute for Health Development | 770 | 9^th^ August 2021 |
| Iran | Iran University of Medical Sciences, Tehran, Iran | IR.IUMS.REC.1400.758 | 21^st^ November 2021 |
| Lebanon | Institutional Review Board,American University of Beirut | PED.KY.01 | 13^th^ July 2021 |
| Malaysia | Medical Research & Ethics Committee, Ministry of Health Malaysia | KKM/NIHSEC/ P21-718 ( 4 ) | 5^th^ May 2021 |
| Mexico | Centre of Investigation in Health Sciences, Anahuac University, Mexico | 202214 | 31^st^ March 2022 |
| Qatar | Medical Research Center, Hamad Medical Corporation, Doha-Qatar | MRC-01-21-277 | 25^th^ April 2021 |
| UK_England and Wales | 1. National Information Governance Board  2. Confidentiality Advisory Group of the Health Research Authority  3. Health & Social Care Information Centre (HSCIC), Data Access Advisory Group | 1. ECC 5-05 (f)/2012  2. 15/CAG/0119  3. DARS-NIC-359651-H3R1P-v5.2. | 1. From 10^th^ October 2012  2. From 1^st^ May 2015 |
| UK_Scotland | Public Health Scotland | 20210218-VulnerableNewbornMeasurement | 30^th^ March 2021 |
| **Exemptions (*e.g., IRB approval not required since existing ethics approval in place, etc)*** | | | |
| Argentina, Chile, Czech Republic, Denmark, Finland, Netherlands, Peru, South Korea, Sweden, N. Ireland, Uruguay, USA publicly available data from <https://www.cdc.gov/nchs/data_access/Vitalstatsonline.htm> | | | |

# **S3: Definitions**

| **Definitions** |  |
| --- | --- |
| Livebirth | Is the complete expulsion or extraction from a woman of a fetus, irrespective of the duration of the pregnancy, which, after such separation, shows signs of life |
| Birthweight | Is defined as the weight of the fetus or newborn obtained immediately after birth. For livebirths, measurement of birthweight within the first hour of life before significant postnatal weight loss has occurred is preferable. If the birth weight was measured repeatedly, the median value will be used. Birthweight can be measured using digital or analogue scales. For the purposes of this work, weights of newborns taken at ≥72 hours after birth will be excluded. |
| Gestational Age | The duration of gestation measured from the first day of the last menstrual period (LMP). Gestational age will be analysed in days where possible. Gestational age measured by LMP, early pregnancy ultrasound or best obstetric estimate (BEO) will be included |
| Neonatal death | A neonatal death is defined as a death during the first 28 days after live birth (days 0-27). An early neonatal death is a death during the first 7 days after live birth (days 0 – 6), a late neonatal death is a death day 7 – 27 after a livebirth. |
| Stillbirth: | A stillbirth is ‘the complete expulsion or extraction from a woman of a fetus following a fetal death at 22 or more completed weeks of gestation; or if gestational age is not available with a birthweight of 500 grams or more’. |
| **Calculated variables** |  |
| Preterm birth | A birth before 37 completed weeks of gestation (or before 259 days of gestation) as measured from the first day of the last menstrual period (LMP) or by early ultrasound. |
| Term birth | A birth from 37 completed weeks of gestation as measured from the first day of the last menstrual period (LMP) or by early ultrasound. |
| Low birthweight | A birth with birthweight of less than 2,500grams |
| Non-Low birthweight | A birth with birthweight of ≥2,500grams |
| Small for gestational age | A birth with a birthweight for gestational age and sex of <10th centile according to INTERGROWTH-21st international standards |
| Appropriate for gestational age | A birth with a birthweight for gestational age and sex from 10th to 90th centiles according to INTERGROWTH-21st international standards |
| Large for gestational age | A birth with a birthweight for gestational age and sex of >90th centile according to INTERGROWTH-21st international standards |

Sources ^2, 3^

# **S4: Newborn types based on birthweight, gestational age, and size for gestational age** **according to INTERGROWTH-21st newborn size standards.**

## S4a: Methods used to extend INTERGROWTH-21st newborn size standards from 22^+0^ to 44^+6^

For the purpose of this analysis, the published INTERGROWTH-21^st^ standards were extrapolated from the original models that covered the period from 24^+0^ to 42^+6^ by extending the range of gestational age starting from 22^+0^ to 23^+6^ weeks and from 43^+0^ to 44^+6^


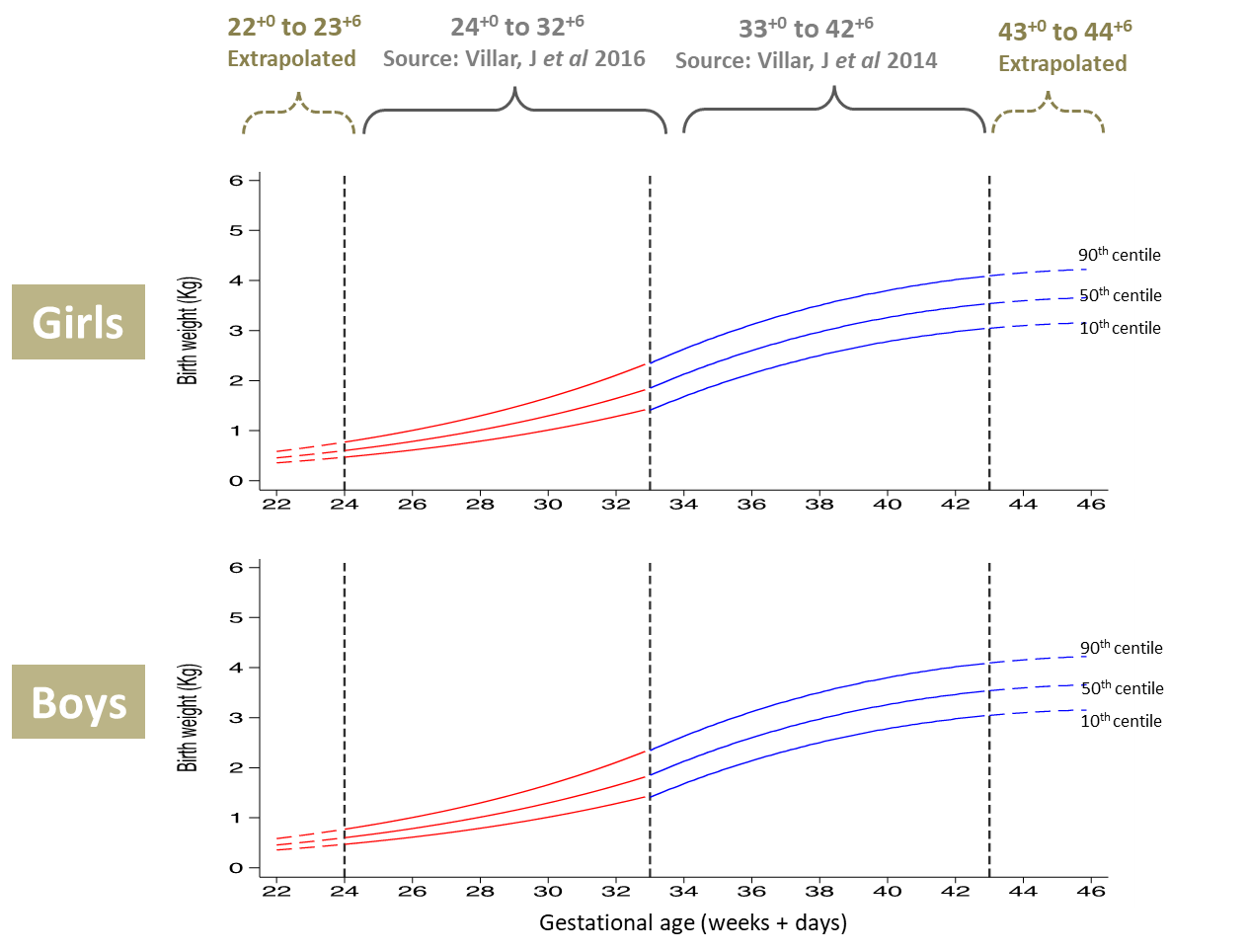


Sources: ^4-6^

## S4b: Figure illustrating original newborn types

The original newborn types suggested by Ashorn et al ^7^


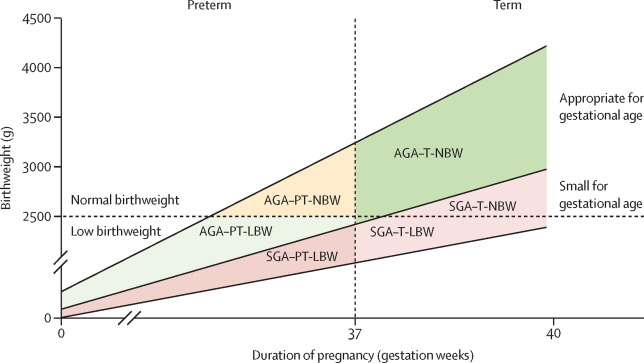


# **S5: Additional information on input data**

## S5a: Flowchart of database construction.

**
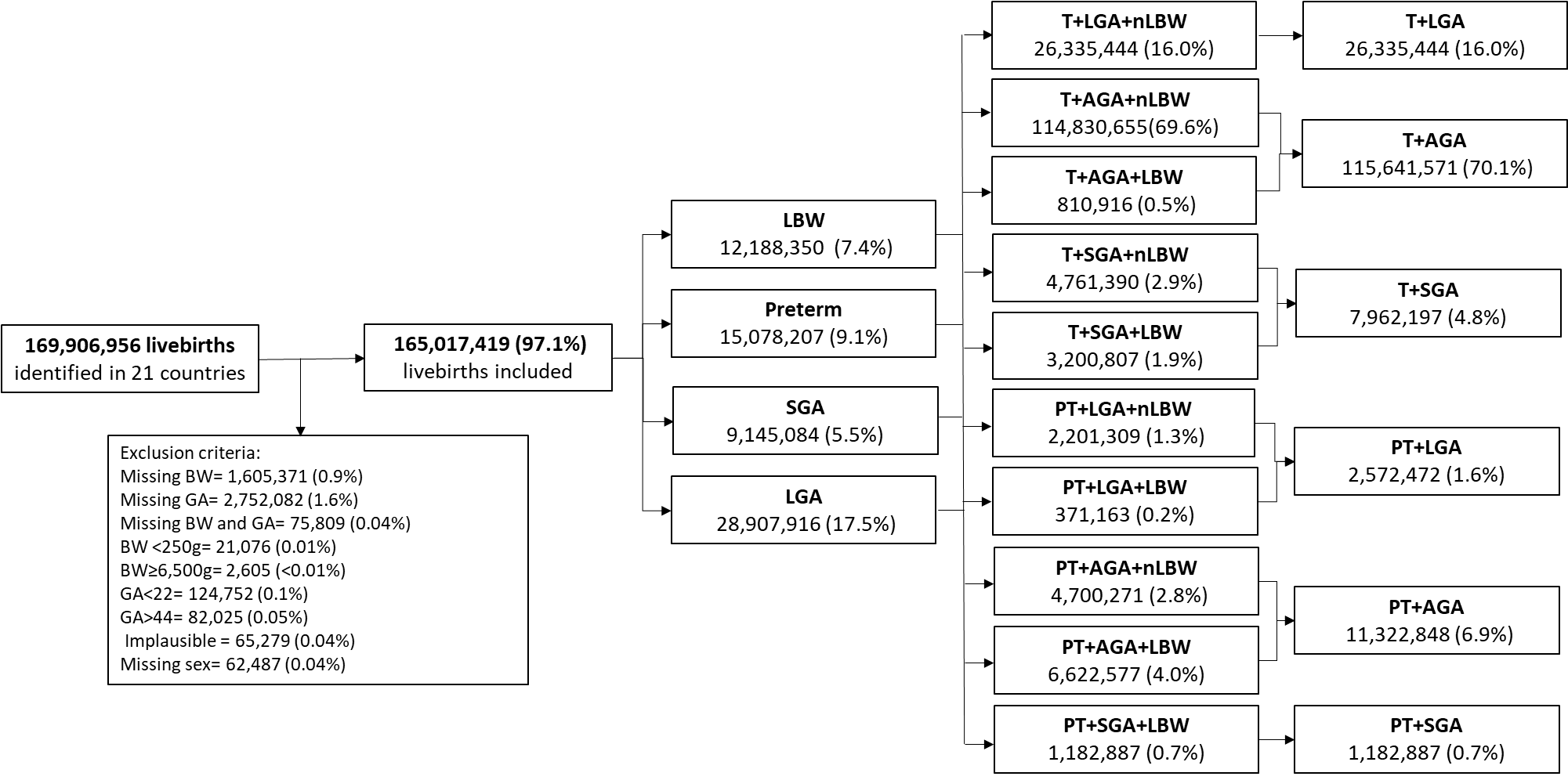
**

## S5b: Maternal baseline characteristics of livebirths in 21 participant countries, by SDG Regions

Baseline characteristics are available for 2014 onwards in the US and not available in Canada

Multiple pregnancies in Argentina are reported as single or multiple, then the category "twins" includes all multiple pregnancies

Births outside health facilities in the Netherlands include a combination of outpatient births attended in hospitals or at home supervised by primary care midwifes

| **Countries/SDG Region** | **Eastern, South Eastern Asia and Oceania** | | **Latin America and the Caribbean** | | | | | | **Northern America, Australia and New Zealand, Central Asia and Europe** | | | | | | | | | | | **Western Asia and Northern Africa** | | |
| --- | --- | --- | --- | --- | --- | --- | --- | --- | --- | --- | --- | --- | --- | --- | --- | --- | --- | --- | --- | --- | --- | --- |
|  | South Korea | Malaysia | Argentina^a^ | Brazil | Chile | Mexico | Peru | Uruguay | Australia | Czech Republic | Denmark | England & Wales | Estonia | Finland | Netherlands | N. Ireland | Scotland | Sweden | USA | Iran | Lebanon | Qatar |
|  | % | % | % | % | % | % | % | % | % | % | % | % | % | % | % | % | % | % | % | % | % | % |
| Sex |  |  |  |  |  |  |  |  |  |  |  |  |  |  |  |  |  |  |  |  |  |  |
| Male | 51.4 | 42.5 | 50.0 | 46.6 | 50.9 | 48.4 | 51.1 | 50.4 | 51.3 | 49.9 | 50.2 | 51.2 | 51.6 | 51.8 | 50.8 | 51.5 | 51.1 | 51.4 | 50.8 | 11.4 | 47.7 | 50.2 |
| Female | 48.6 | 39.5 | 47.8 | 44.4 | 48.8 | 45.4 | 48.8 | 48.3 | 48.5 | 47.3 | 47.6 | 48.8 | 48.4 | 49.4 | 48.3 | 48.4 | 48.5 | 48.6 | 48.5 | 10.6 | 44.6 | 48.2 |
| Schooling |  |  |  |  |  |  |  |  |  |  |  |  |  |  |  |  |  |  |  |  |  |  |
| Primary & Secondary | 1.9 | 0.0 | 43.0 | 0.0 | 15.5 | 53.2 | 34.0 | 3.3 | 0.0 | 11.2 | 17.4 | 0.0 | 29.5 | 36.8 | 0.0 | 0.0 | 0.0 | 19.1 | 13.1 | 9.5 | 31.0 | 0.0 |
| Upper secondary & professional | 23.9 | 0.0 | 36.2 | 0.0 | 57.1 | 25.5 | 57.0 | 0.2 | 0.0 | 22.1 | 42.0 | 0.0 | 22.9 | 7.4 | 0.0 | 0.0 | 0.0 | 40.1 | 53.2 | 7.6 | 23.1 | 0.0 |
| Bachelors and above | 73.5 | 0.0 | 13.9 | 0.0 | 27.0 | 13.8 | 8.9 | 0.3 | 0.0 | 28.1 | 32.2 | 0.0 | 47.5 | 22.9 | 0.0 | 0.0 | 0.0 | 39.9 | 31.6 | 4.8 | 27.7 | 0.0 |
| Missing | 0.8 | 100.0 | 4.7 | 100 | 0.2 | 1.4 | 0.0 | 0.0 | 100 | 35.8 | 6.2 | 100 | 0.1 | 34.1 | 99.1 | 100 | 100 | 1.0 | 2.1 | 0.2 | 10.6 | 100.0 |
| Maternal age (in years) |  |  |  |  |  |  |  |  |  |  |  |  |  |  |  |  |  |  |  |  |  |  |
| ≤19 | 0.5 | 3.2 | 11.9 | 16.3 | 14.3 | 18.6 | 12.6 | 15.9 | 3.6 | 2.0 | 1.2 | 3.1 | 2.1 | 2.5 | 1.0 | 3.7 | 6.0 | 1.4 | 11.9 | 1.2 | 3.9 | 6.0 |
| 20 to 34 | 76.2 | 65.5 | 66.8 | 62.9 | 68.9 | 66.1 | 68.6 | 67.7 | 74.5 | 74.3 | 77.4 | 74.4 | 75.3 | 79.3 | 76.9 | 75.8 | 73.7 | 77.4 | 73.1 | 16.0 | 67.0 | 77.3 |
| ≥35 | 23.2 | 13.4 | 17.6 | 11.8 | 16.5 | 8.9 | 18.7 | 15.0 | 21.8 | 21.0 | 19.1 | 22.5 | 22.5 | 19.4 | 20.9 | 20.4 | 20.1 | 21.2 | 14.3 | 4.8 | 14.5 | 15.1 |
| Missing | 0.1 | 0.0 | 1.4 | 0.0 | 0.0 | 0.3 | 0.0 | 0.1 | 0.0 | 0.0 | 0.0 | 0.0 | 0.0 | 0.0 | 0.3 | 0.0 | 0.0 | 0.0 | 0.0 | 0.0 | 6.8 | 0.0 |
| Place of delivery |  |  |  |  |  |  |  |  |  |  |  |  |  |  |  |  |  |  |  |  |  |  |
| Outside health facility | 0.0 | 0.0 | 0.3 | 0.0 | 0.4 | 1.1 | 0.9 | 0.0 | 1.0 | 0.3 | 0.0 | 0.0 | 0.7 | 0.3 | 26.7 | 0.6 | 0.7 | 0.0 | 1.2 | 0.2 | 7.7 | 1.0 |
| Health facility | 0.0 | 82.1 | 96.2 | 0.0 | 99.4 | 77.4 | 99.1 | 0.0 | 99.0* | 95.3 | 0.0 | 0.0 | 99.3 | 99.4 | 72.0 | 99.2 | 99.0 | 99.9 | 98.2 | 21.8 | 92.3 | 99.0 |
| Missing | 100.0 | 0.0 | 1.2 | 100.0 | 0.0 | 15.3 | 0.0 | 100.0 | 0.0 | 1.6 | 100.0 | 100.0 | 0.0 | 0.0 | 0.4 | 0.0 | 0.0 | 0.1 | 0.0 | 0.1 | 0.0 | 0.0 |
| Mode of delivery |  |  |  |  |  |  |  |  |  |  |  |  |  |  |  |  |  |  |  |  |  |  |
| Vaginal | 0.0 | 56.7 | 0.0 | 40.0 | 0.0 | 50.8 | 63.4 | 53.6 | 67.8 | 71.8 | 77.2 | 0.0 | 79.8 | 82.7 | 79.6 | 68.2 | 71.3 | 82.5 | 68.8 | 22.0 | 51.0 | 64.5 |
| Caesarean section | 0.0 | 22.1 | 0.0 | 50.9 | 0.0 | 42.8 | 36.1 | 42.7 | 32.1 | 23.8 | 20.6 | 0.0 | 20.2 | 16.9 | 16.4 | 31.7 | 28.3 | 17.5 | 30.3 | 0.0 | 35.7 | 33.5 |
| Missing | 100.0 | 0.0 | 97.8 | 0.1 | 100.0 | 0.2 | 0.5 | 2.4 | 0.0 | 1.6 | 0.0 | 100.0 | 0.0 | 0.0 | 3.1 | 0.0 | 0.0 | 0.0 | 0.1 | 0.0 | 5.7 | 0.3 |
| Parity (number of pregnancies) |  |  |  |  |  |  |  |  |  |  |  |  |  |  |  |  |  |  |  |  |  |  |
| 1 | 52.0 | 0.0 | 32.2 | 0.0 | 42.9 | 34.2 | 31.7 | 0.0 | 41.6 | 46.4 | 43.8 | 0.0 | 38.4 | 41.5 | 44.1 | 29.5 | 44.2 | 43.3 | 35.0 | 22.0 | 32.1 | 0.0 |
| 2 | 37.7 | 28.4 | 26.7 | 0.0 | 32.6 | 28.1 | 28.5 | 0.0 | 33.6 | 36.6 | 35.4 | 0.0 | 36.8 | 33.5 | 35.4 | 19.3 | 34.2 | 36.7 | 29.7 | 0.0 | 27.4 | 0.0 |
| ≥3 | 10.0 | 53.4 | 32.5 | 0.0 | 24.3 | 31.5 | 38.9 | 0.0 | 23.0 | 14.2 | 17.5 | 0.0 | 24.8 | 24.6 | 19.1 | 20.1% | 20.5 | 20.0 | 34.7 | 0.0 | 30.6 | 0.0 |
| Missing | 0.3 | 0.3 | 6.4 | 100.0 | 0.0 | 0.1 | 0.9 | 100.0 | 1.5 | 0.0 | 1.1 | 100.0 | 0.0 | 0.0 | 0.4 | 31.0 | 0.8 | 0.0 | 0.0 | 100.0 | 2.2 | 100.0 |
| Number of babies |  |  |  |  |  |  |  |  |  |  |  |  |  |  |  |  |  |  |  |  |  |  |
| Singleton | 96.4 | 80.3 | 95.6 | 89.0 | 97.8 | 92.2 | 98.0 | 95.5 | 96.8 | 94.7 | 93.9 | 97.0 | 98.4 | 96.8 | 96.0 | 96.9 | 96.7 | 97.2 | 96.0 | 21.3 | 86.8 | 93.0 |
| Twins | 3.4 | 1.7 | 2.0 | 1.8 | 1.9 | 1.3 | 1.8 | 1.9 | 3.0 | 2.5 | 3.9 | 2.9 | 1.6 | 2.8 | 3.1 | 2.9 | 2.9 | 2.8 | 3.2 | 0.7 | 3.4 | 3.8 |
| Triplets or higher | 0.1 | 0.0 | 0.0 | 0.0 | 0.1 | 0.0 | 0.0 | 1.3 | 0.1 | 0.0 | 0.0 | 0.1 | 0.0 | 0.0 | 0.1 | 0.1 | 0.1 | 0.1 | 0.1 | 0.0 | 0.4 | 0.4 |
| Missing | 0.1 | 0.0 | 0.2 | 0.1 | 0.0 | 0.3 | 0.1 | 0.0 | 0.0 | 0.0 | 0.0 | 0.0 | 0.0 | 0.0 | 0.0 | 0.0 | 0.0 | 0.0 | 0.0 | 0.0 | 1.7 | 1.2 |
| Gestational age assessment |  |  |  |  |  |  |  |  |  |  |  |  |  |  |  |  |  |  |  |  |  |  |
| Ultrasound before 14 weeks | 0.0 | 0.0 | 0.0 | 0.0 | 0.0 | 0.0 | 0.0 | 0.0 | 0.0 | 0.0 | 0.0 | 0.0 | 83.3 | 0.0 | 0.0 | 0.0 | 0.0 | 90.2 | 0.0 | 0.0 | 0.0 | 0.0 |
| Ultrasound 14 to 24 weeks | 0.0 | 0.0 | 0.0 | 0.0 | 0.0 | 0.0 | 0.0 | 0.0 | 0.0 | 0.0 | 0.0 | 0.0 | 0.0 | 0.0 | 0.0 | 0.0 | 0.0 | 0.0 | 0.0 | 0.0 | 0.0 | 0.0 |
| Ultrasound after 24 weeks | 0.0 | 0.0 | 0.0 | 0.0 | 0.0 | 0.0 | 0.0 | 0.0 | 0.0 | 0.0 | 0.0 | 0.0 | 14.5 | 0.0 | 0.0 | 0.0 | 0.0 | 0.0 | 0.0 | 0.0 | 0.0 | 0.0 |
| Ultrasound timing unknown | 0.0 | 0.0 | 0.0 | 0.0 | 0.0 | 0.0 | 0.0 | 0.0 |  | 2.5 | 0.0 | 0.0 | 0.0 | 0.0 | 0.0 | 0.0 | 0.0 | 0.0 | 0.0 | 0.0 | 0.0 | 0.0 |
| Best obstetric estimate | 0.0 | 0.0 | 0.0 | 0.0 | 0.0 | 0.0 | 0.0 | 0.0 | 0.0 | 0.0 | 0.0 | 0.0 | 1.6 | 99.6 | 0.0 | 0.0 | 100 | 0.0 | 94.8 | 0.0 | 0.0 | 0.0 |
| Last menstrual period | 0.0 | 0.0 | 0.0 | 0.0 | 0.0 | 0.0 | 0.0 | 0.0 | 0.0 | 94.7 | 100 | 0.0 | 0.0 | 0.0 | 0.0 | 0.0 | 0.0 | 0.0 | 4.3 | 0.0 | 0.0 | 0.0 |
| Symphysis fundal height | 0.0 | 0.0 | 0.0 | 0.0 | 0.0 | 0.0 | 0.0 | 0.0 | 0.0 | 0.0 | 0.0 | 0.0 | 0.0 | 0.0 | 0.0 | 0.0 | 0.0 | 0.0 | 0.0 | 0.0 | 0.0 | 0.0 |
| Method not stated | 100 | 100 | 97.8 | 100 | 100 | 93.8 | 99.9 | 100 | 100 | 0.0 | 0.0 | 100 | 0.7 | 0.0 | 99.1 | 100 | 0.0 | 0.0 | 0.2 | 100 | 92.3 | 100 |

*Data available only for 2016 - 2020

# **S6: Additional analyses and results**

## S6a: Preterm, LBW, SGA, and LGA amongst 165,017,419 livebirths included from 23 countries, by SDG Region


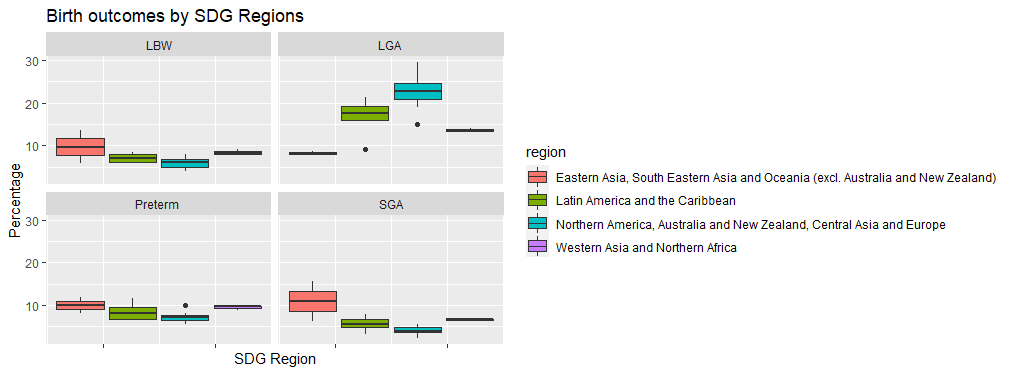


Box plots summarize the median and interquantile range (25^th^ and 75^th^ percentiles)

## S6b: Prevalence of six newborn types among livebirths (n: 165,017,419), by SDG Region

| **Region/Country** | **Total livebirths** | **PT+SGA** | **PT+AGA** | **PT+LGA** | **T+SGA** | **T+AGA** | **T+LGA** |
| --- | --- | --- | --- | --- | --- | --- | --- |
|  | **n** | **%** | **%** | **%** | **%** | **%** | **%** |
| **Eastern Asia, South Eastern Asia and Oceania** |  |  |  |  |  |  |  |
| South Korea | 4,050,568 | 0.6 | 6.7 | 0.7 | 5.6 | 79.6 | 7.9 |
| Malaysia | 888,165 | 1.5 | 8.0 | 2.3 | 14.2 | 68.4 | 5.6 |
| **Latin America and the Caribbean** |  |  |  |  |  |  |  |
| Argentina | 1,238,453 | 0.9 | 7.0 | 1.7 | 4.7 | 67.9 | 17.8 |
| Brazil | 21,325,177 | 0.9 | 7.0 | 3.7 | 7.1 | 68.8 | 12.5 |
| Chile | 4,320,964 | 0.6 | 5.7 | 0.9 | 2.6 | 69.9 | 20.4 |
| Mexico | 23,414,789 | 0.6 | 5.3 | 0.5 | 6.5 | 78.4 | 8.7 |
| Peru | 2,931,061 | 0.7 | 5.2 | 0.8 | 4.6 | 73.6 | 15.2 |
| Uruguay | 492,825 | 0.7 | 7.4 | 1.0 | 3.8 | 69.3 | 17.8 |
| **N. America, Australia and NZ, Central Asia and Europe** |  |  |  |  |  |  |  |
| Australia | 5,746,938 | 0.6 | 6.1 | 1.0 | 3.3 | 67.8 | 21.2 |
| Canada* | 4,118,661 | 0.6 | 6.4 | 1.1 | 3.2 | 67.7 | 21.0 |
| Czech Republic | 109,131 | 0.6 | 5.7 | 0.6 | 4.8 | 73.9 | 14.4 |
| Denmark | 1,100,854 | 0.5 | 5.2 | 1.0 | 2.9 | 64.5 | 25.9 |
| England & Wales | 3,212,492 | 0.9 | 5.9 | 0.8 | 4.2 | 70.1 | 18.2 |
| Estonia | 82,427 | 0.4 | 4.6 | 0.8 | 1.9 | 63.5 | 28.8 |
| Finland | 1,122,157 | 0.6 | 4.4 | 0.6 | 3.3 | 68.2 | 22.9 |
| Netherlands | 1,844,765 | 0.6 | 5.6 | 0.8 | 3.1 | 67.0 | 23.0 |
| Northern Ireland | 155,779 | 0.6 | 5.8 | 1.0 | 2.5 | 65.4 | 24.8 |
| Scotland | 1,124,505 | 0.7 | 6.0 | 0.9 | 4.5 | 67.4 | 20.5 |
| Sweden | 2,101,407 | 0.5 | 4.7 | 0.6 | 3.3 | 67.2 | 23.7 |
| USA | 80,193,986 | 0.7 | 7.7 | 1.6 | 4.1 | 67.8 | 18.1 |
| **Western Asia and Northern Africa** |  |  |  |  |  |  |  |
| Iran | 5,045,747 | 0.8 | 6.6 | 1.4 | 5.5 | 73.8 | 11.9 |
| Lebanon | 302,231 | 0.9 | 7.2 | 1.6 | 5.8 | 72.7 | 11.9 |
| Qatar | 94,337 | 0.9 | 7.7 | 1.4 | 5.7 | 71.6 | 12.7 |

* Does not include Quebec due to different data collection system

## S6c: Six newborn types sorted in ascending order (n: 165,017,419)

| **PT+SGA** | | **PT+AGA** | | **PT+LGA** | | **T+SGA** | | **T+AGA** | | **T+LGA** | |
| --- | --- | --- | --- | --- | --- | --- | --- | --- | --- | --- | --- |
| Country | % | Country | % | Country | % | Country | % | Country | % | Country | % |
| Estonia | 0.4 | Finland | 4.4 | Mexico | 0.5 | Estonia | 1.9 | Estonia | 63.5 | Malaysia | 5.6 |
| Denmark | 0.5 | Estonia | 4.6 | Czech Republic | 0.6 | Northern Ireland | 2.5 | Denmark | 64.5 | South Korea | 7.9 |
| Sweden | 0.5 | Sweden | 4.7 | Finland | 0.6 | Chile | 2.6 | Northern Ireland | 65.4 | Mexico | 8.7 |
| Finland | 0.6 | Peru | 5.2 | Sweden | 0.6 | Denmark | 2.9 | Netherlands | 67.0 | Iran | 11.9 |
| Netherlands | 0.6 | Denmark | 5.2 | South Korea | 0.7 | Netherlands | 3.1 | Sweden | 67.2 | Lebanon | 11.9 |
| Australia | 0.6 | Mexico | 5.3 | England & Wales | 0.8 | Canada | 3.2 | Scotland | 67.4 | Brazil | 12.5 |
| Northern Ireland | 0.6 | Netherlands | 5.6 | Peru | 0.8 | Sweden | 3.3 | Canada | 67.7 | Qatar | 12.7 |
| Chile | 0.6 | Czech Republic | 5.7 | Netherlands | 0.8 | Australia | 3.3 | Australia | 67.8 | Czech Republic | 14.4 |
| Canada | 0.6 | Chile | 5.7 | Estonia | 0.8 | Finland | 3.3 | USA | 67.8 | Peru | 15.2 |
| Mexico | 0.6 | Northern Ireland | 5.8 | Scotland | 0.9 | Uruguay | 3.8 | Argentina | 67.9 | Uruguay | 17.8 |
| South Korea | 0.6 | England & Wales | 5.9 | Chile | 0.9 | USA | 4.1 | Finland | 68.2 | Argentina | 17.8 |
| Czech Republic | 0.6 | Scotland | 6.0 | Australia | 1.0 | England & Wales | 4.2 | Malaysia | 68.4 | USA | 18.1 |
| Peru | 0.7 | Australia | 6.1 | Northern Ireland | 1.0 | Scotland | 4.5 | Brazil | 68.8 | England & Wales | 18.2 |
| Scotland | 0.7 | Canada | 6.4 | Denmark | 1.0 | Peru | 4.6 | Uruguay | 69.3 | Chile | 20.4 |
| Uruguay | 0.7 | Iran | 6.6 | Uruguay | 1.0 | Argentina | 4.7 | Chile | 69.9 | Scotland | 20.5 |
| USA | 0.7 | South Korea | 6.7 | Canada | 1.1 | Czech Republic | 4.8 | England & Wales | 70.1 | Canada | 21.0 |
| Iran | 0.8 | Argentina | 7.0 | Iran | 1.4 | Iran | 5.5 | Qatar | 71.6 | Australia | 21.2 |
| England & Wales | 0.9 | Brazil | 7.0 | Qatar | 1.4 | South Korea | 5.6 | Lebanon | 72.7 | Finland | 22.9 |
| Brazil | 0.9 | Lebanon | 7.2 | Lebanon | 1.6 | Qatar | 5.7 | Peru | 73.6 | Netherlands | 23.0 |
| Lebanon | 0.9 | Uruguay | 7.4 | USA | 1.6 | Lebanon | 5.8 | Iran | 73.8 | Sweden | 23.7 |
| Qatar | 0.9 | USA | 7.7 | Argentina | 1.7 | Mexico | 6.5 | Czech Republic | 73.9 | Northern Ireland | 24.8 |
| Argentina | 0.9 | Qatar | 7.7 | Malaysia | 2.3 | Brazil | 7.1 | Mexico | 78.4 | Denmark | 25.9 |
| Malaysia | 1.5 | Malaysia | 8.0 | Brazil | 3.7 | Malaysia | 14.2 | South Korea | 79.6 | Estonia | 28.8 |
|  |  |  |  |  |  |  |  |  |  |  |  |
| **Region key** | | | | |  |  |  |  |  |  |  |
| Eastern Asia, South Eastern Asia and Oceania | | | | |  |  |  |  |  |  |  |
| Latin America and the Caribbean | | | | |  |  |  |  |  |  |  |
| Northern America, Australia and New Zealand, Central Asia and Europe | | | | |  |  |  |  |  |  |  |
| Western Asia and Northern Africa | | | | |  |  |  |  |  |  |  |

## S6d: Ten newborn types amongst livebirths (n=165,017,419), by country

| **Region**  **/Country** | **Total livebirths** | **PT+SGA+LBW** | **PT+AGA+LBW** | **PT+AGA+non-LBW** | **PT+LGA+LBW** | **PT+LGA+non-LBW** | **T+SGA+LBW** | **T+SGA+non-LBW** | **T+AGA+LBW** | **T+AGA+non-LBW** | **T+LGA+non-LBW** |
| --- | --- | --- | --- | --- | --- | --- | --- | --- | --- | --- | --- |
|  | **n** | **%** | **%** | **%** | **%** | **%** | **%** | **%** | **%** | **%** | **%** |
| **Eastern Asia, South Eastern Asia and Oceania** | | | | | | | | | | | |
| South Korea | 4,050,568 | 0.6 | 3.1 | 3.6 | 0.1 | 0.6 | 1.6 | 4.0 | 0.5 | 78.0 | 7.9 |
| Malaysia | 888,165 | 1.5 | 4.6 | 3.4 | 0.3 | 2.0 | 6.2 | 8.0 | 0.9 | 67.5 | 5.6 |
| **Latin America and the Caribbean** | | | | | | | | | | | |
| Argentina | 1,238,453 | 0.9 | 4.1 | 2.9 | 0.2 | 1.5 | 2.3 | 2.4 | 0.4 | 67.5 | 17.8 |
| Brazil | 21,325,177 | 0.9 | 3.8 | 3.2 | 0.3 | 3.4 | 2.9 | 4.2 | 0.5 | 68.4 | 12.5 |
| Chile | 4,320,964 | 0.6 | 3.4 | 2.3 | 0.2 | 0.7 | 1.3 | 1.3 | 0.4 | 69.5 | 20.4 |
| Mexico | 23,414,789 | 0.6 | 3.3 | 2.1 | 0.1 | 0.4 | 1.5 | 5.0 | 0.6 | 77.8 | 8.7 |
| Peru | 2,931,061 | 0.7 | 3.1 | 2.1 | 0.2 | 0.6 | 1.9 | 2.7 | 0.4 | 73.2 | 15.2 |
| Uruguay | 492,825 | 0.7 | 4.4 | 3.0 | 0.2 | 0.8 | 1.9 | 1.9 | 0.5 | 68.8 | 17.8 |
| **Northern America, Australia and NZ, Central Asia and Europe** | | | | | | | | | | | |
| Australia | 5,746,938 | 0.6 | 3.6 | 2.5 | 0.2 | 0.8 | 1.5 | 1.8 | 0.4 | 67.4 | 21.2 |
| Canada* | 4,118,661 | 0.6 | 3.7 | 2.7 | 0.2 | 0.9 | 1.4 | 1.8 | 0.4 | 67.3 | 21.0 |
| Czech Republic | 109,131 | 0.6 | 3.6 | 2.1 | 0.1 | 0.4 | 1.9 | 2.9 | 0.5 | 73.4 | 14.4 |
| Denmark | 1,100,854 | 0.5 | 3.1 | 2.1 | 0.2 | 0.8 | 1.2 | 1.7 | 0.3 | 64.2 | 25.9 |
| England & Wales | 3,212,492 | 0.9 | 3.7 | 2.2 | 0.1 | 0.6 | 1.9 | 2.2 | 0.7 | 69.4 | 18.2 |
| Estonia | 82,427 | 0.4 | 2.6 | 2.0 | 0.2 | 0.6 | 0.8 | 1.1 | 0.3 | 63.3 | 28.8 |
| Finland | 1,122,157 | 0.6 | 2.3 | 2.1 | 0.1 | 0.5 | 1.1 | 2.3 | 0.1 | 68.1 | 22.9 |
| Netherlands | 1,844,765 | 0.6 | 3.3 | 2.3 | 0.2 | 0.6 | 1.4 | 1.6 | 0.5 | 66.5 | 23.0 |
| Northern Ireland | 155,779 | 0.6 | 3.5 | 2.3 | 0.2 | 0.8 | 1.2 | 1.3 | 0.5 | 64.9 | 24.8 |
| Scotland | 1,124,505 | 0.7 | 3.8 | 2.3 | 0.2 | 0.7 | 1.9 | 2.6 | 0.4 | 67.0 | 20.5 |
| Sweden | 2,101,407 | 0.5 | 2.4 | 2.2 | 0.1 | 0.5 | 1.0 | 2.3 | 0.1 | 67.1 | 23.7 |
| USA | 80,193,986 | 0.7 | 4.6 | 3.1 | 0.3 | 1.3 | 1.9 | 2.1 | 0.5 | 67.3 | 18.1 |
| **Western Asia and Northern Africa** | | | | | | | | | | | |
| Iran | 5,045,747 | 0.8 | 3.8 | 2.8 | 0.3 | 1.1 | 2.5 | 3.0 | 0.4 | 73.5 | 11.9 |
| Lebanon | 302,231 | 0.9 | 3.8 | 3.4 | 0.2 | 1.4 | 2.8 | 2.9 | 0.5 | 72.1 | 11.9 |
| Qatar | 94,337 | 0.9 | 4.8 | 2.9 | 0.3 | 1.1 | 2.5 | 3.1 | 0.7 | 71.0 | 12.7 |

* Hospital births; the province of Quebec is not included due to different data collection system

##

## S6e: Sensitivity analysis for six newborn Types prevalence excluding years with missing gestational age >20%.

Malaysia excluding 3 country-years of data (2010, 2011 and 2017). Brazil excluding 1 country-years of data (2011)

| **Country /Period of observation** | **Live births** | **PT+SGA** | **PT+AGA** | **PT+LGA** | **T+SGA** | **T+AGA** | **T+LGA** |
| --- | --- | --- | --- | --- | --- | --- | --- |
|  | **(n)** | **(%)** | **(%)** | **(%)** | **(%)** | **(%)** | **(%)** |
| Malaysia |  |  |  |  |  |  |  |
| 2010-2017 | 888,165 | 1.5 | 8.0 | 2.3 | 14.2 | 68.4 | 5.6 |
| 2012-2016 | 607,021 | 1.5 | 8.0 | 2.1 | 14.3 | 68.7 | 5.5 |
| Brazil |  |  |  |  |  |  |  |
| 2011-2018 | 21,325,177 | 0.9 | 7.0 | 3.7 | 7.1 | 68.8 | 12.5 |
| 2012-2018 | 19,760,732 | 0.9 | 7.0 | 3.6 | 7.0 | 69.0 | 12.6 |

## S6f: Three-year rolling average changes for Vulnerable Newborn (VN) Types in 19 countries*, 2000 to 2009 vs 2010 to 2021

| **Country** | **2000-2009** | | | | | | **2010-2021** | | | | | |
| --- | --- | --- | --- | --- | --- | --- | --- | --- | --- | --- | --- | --- |
|  | **Years** | **Livebirths** | **Small vulnerable newborns** | | **Large vulnerable newborns**  **(T+AGA)** | | **Years** | **Livebirths** | **Small vulnerable newborns** | | **Large vulnerable newborns**  **(T+AGA)** | |
|  |  |  | **Number of years with increases** | **Number of years with decreases** | **Number of years with increases** | **Number of years with decreases** |  |  | **Number of years with increases** | **Number of years with decreases** | **Number of years with increases** | **Number of years with decreases** |
| **South Korea** |  |  |  |  |  |  | 10 | 4,116,808 | 0 | 0 | 0 | 0 |
| **Malaysia** |  |  |  |  |  |  | 8 | 888,165 | 0 | 0 | 0 | 0 |
| **Brazil** |  |  |  |  |  |  | 8 | 21,325,177 | 0 | 2 | 0 | 0 |
| **Chile** | 10 | 2,395,497 | 0 | 0 | 0 | 0 | 8 | 1,925,467 | 0 | 0 | 0 | 0 |
| **Mexico** | 2 | 3,748,471 | 0 | 0 | 0 | 0 | 10 | 19,666,318 | 0 | 0 | 0 | 0 |
| **Peru** |  |  |  |  |  |  | 8 | 2,931,061 | 0 | 0 | 0 | 1 |
| **Uruguay** | 1 | 39,938 | 0 | 0 | 0 | 0 | 11 | 452,887 | 0 | 0 | 1 | 0 |
| **Australia** | 10 | 2,701,836 | 0 | 0 | 0 | 0 | 10 | 3,045,102 | 0 | 0 | 0 | 0 |
| **Canada**** | 5 | 1,315,266 | 0 | 0 | 0 | 0 | 10 | 2,803,395 | 0 | 0 | 0 | 0 |
| **Denmark** | 10 | 636,237 | 0 | 0 | 0 | 2 | 8 | 464,617 | 0 | 0 | 0 | 0 |
| **England & Wales** |  |  |  |  |  |  | 5 | 3,212,492 | 0 | 0 | 0 | 0 |
| **Estonia** |  |  |  |  |  |  | 6 | 82,427 | 0 | 0 | 0 | 0 |
| **Finland** | 10 | 572,969 | 0 | 0 | 0 | 2 | 10 | 549,188 | 0 | 0 | 0 | 0 |
| **Netherlands** |  |  |  |  |  |  | 11 | 1,844,765 | 0 | 0 | 0 | 0 |
| **Scotland** | 10 | 537,517 | 0 | 0 | 0 | 0 | 11 | 586,988 | 0 | 0 | 0 | 0 |
| **Sweden** | 10 | 976,673 | 0 | 0 | 0 | 1 | 10 | 1,124,734 | 0 | 0 | 0 | 0 |
| **USA** | 10 | 41,072,595 | 0 | 0 | 0 | 0 | 10 | 39,121,391 | 0 | 0 | 0 | 0 |
| **Iran** |  |  |  |  |  |  | 5 | 5,045,747 | 0 | 0 | 0 | 0 |
| **Lebanon** | 9 | 112,344 | 0 | 0 | 1 | 1 | 10 | 189,887 | 1 | 0 | 0 | 0 |
| **Qatar** |  |  |  |  |  |  | 4 | 94,337 | 0 | 0 | 0 | 0 |

*Excluding three national datasets for providing pooled data or less than tree disaggregated years. Argentina (data 2017 and 2018), Czech Republic (data 2019), and Northern Ireland (pooled data from 2016 to 2021)

** Years 2005-2019; the province of Quebec is not included due to different data collection system; hospital births

Increases and decreases were absolute changes >0.5% compared to the previous 3-year rolling average period

## S6g: Reporting practices in participating countries

| **Country** | **Units** | **Reporting criteria for very preterm** | | |
| --- | --- | --- | --- | --- |
|  | **Birthweight/ Gestational age** | **Exclusions criteria based on BW** | **Exclusion criteria based on GA** | **Are births following induced Termination of Pregnancy included in the data source?** |
| Argentina | Grams | None | None | No |
|  | Completed weeks |  |  |  |
| Australia | Grams | A small number of births <400 grams are included | A small number of births < 20 weeks are included | Both livebirths and stillbirths may include termination of pregnancy after 20 weeks. |
|  | Completed weeks |  |  |  |
| Brazil | Grams | <350g | <20 weeks | No |
|  | Completed weeks |  |  |  |
| Canada | Grams | None | None | No |
|  | Completed weeks |  |  |  |
| Chile | Grams | None | None | All livebirths are included |
|  | Completed weeks |  |  |  |
| Czech Republic | Grams | None | <22 weeks | No |
|  | Weeks +days |  |  |  |
| Denmark | Grams | None | None | No |
|  | Days |  |  |  |
| England & Wales | Grams | None | 22 weeks | No |
|  | Completed weeks |  |  |  |
| Estonia | Grams | None | <22 weeks | No |
|  | Weeks +days |  |  |  |
| Finland | Grams | None | None | TOPs are possible until 24+0 weeks (confirmed congenital anomaly), but they are not reported in the Medical Birth Register. |
|  | Weeks +days |  |  |  |
| Iran |  |  |  |  |
| South Korea | Grams | None | None | Both livebirths and stillbirths may include termination of pregnancy after 20 weeks. |
| Lebanon | Grams  weeks+days | None | <22 weeks | No |
| Malaysia | Grams | None | None | No |
|  | Completed weeks |  |  |  |
| Mexico | Grams | None | None | No |
|  | Completed weeks |  |  |  |
| Netherlands | Grams | Gestational age ≥22 weeks; if g.a. missing, birthweight ≥500 g | Gestational age ≥22 weeks; if g.a. missing, birthweight ≥500 g | Both livebirths and stillbirths up to 24 weeks gestation may include termination of pregnancy |
|  | Weeks + days |  |  |  |
| Northern Ireland | Grams  Days | None | Upper threshold: Invalid record if gestation at delivery is greater than 44 weeks | Yes. However, until October 2019 abortion was illegal in Northern Ireland and some terminations may have occurred outside the antenatal system in Northern Ireland. |
| Peru | Grams  Days | None | None | All livebirths are included |
| Qatar | Kilograms and grams  Weeks and days | None | None | No |
| Scotland | Grams | None | None | No induced ToPs are included |
|  | Completed weeks |  |  |  |
| Sweden | Grams | None | <23 weeks | Included after 22 weeks |
|  | Days |  |  |  |
| Uruguay | Grams | None | None | No |
|  | Completed weeks |  |  |  |
| United States of America | Grams | None | None | The NCHS recommendation for fetal death definition is to exclude TOPs. Some states do exclude TOPs regardless of gestational age, while some states include TOPs |
|  | Completed weeks |  |  |  |

# **S7. Additional references**

1.Benchimol EI, Smeeth L, Guttmann A, Harron K, Moher D, Petersen I, et al. The REporting of studies Conducted using Observational Routinely-collected health Data (RECORD) statement. PLoS Med. 2015;12(10):e1001885.

2.World Health Organization. ICD-10 Version 2019 2021 [Available from: <https://icd.who.int/browse10/2019/en#/XVII>.

3.World Health Organization. World Health Organization. International Classification of Diseases for Mortality and Morbidity Statistics (ICD-11 MMS) 2018 [ 11th ed:[Available from: [https://icd.who.int/browse11/l-m/en#](https://icd.who.int/browse11/l-m/en)!

4.Villar J, Giuliani F, Fenton TR, Ohuma EO, Ismail LC, Kennedy SH. INTERGROWTH-21st very preterm size at birth reference charts. Lancet. 2016;387(10021):844-5.

5.The International Fetal and Newborn Growth Consortium for the 21st Century. INTERGROWTH-21st 2020 [Available from: <https://intergrowth21.tghn.org/>.

6.Villar J, Cheikh Ismail L, Victora CG, Ohuma EO, Bertino E, Altman DG, et al. International standards for newborn weight, length, and head circumference by gestational age and sex: the Newborn Cross-Sectional Study of the INTERGROWTH-21st Project. Lancet. 2014;384(9946):857-68.

7.Ashorn P, Black RE, Lawn JE, Ashorn U, Klein N, Hofmeyr J, et al. The Lancet Small Vulnerable Newborn Series: science for a healthy start. The Lancet. 2020;396(10253):743-5.
